# Arsenite-Activated JNK Signaling Enhances CPEB4-Vinexin Interaction to Facilitate Stress Granule Assembly and Cell Survival

**DOI:** 10.1371/journal.pone.0107961

**Published:** 2014-09-19

**Authors:** Yu-Wei Chang, Yi-Shuian Huang

**Affiliations:** 1 Institute of Biomedical Sciences, Academia Sinica, Taipei, Taiwan; 2 Graduate Institute of Life Sciences, National Defense Medical Center, Taipei, Taiwan; University of British Columbia, Canada

## Abstract

Stress granules (SGs) are compartmentalized messenger ribonucleoprotein particles (mRNPs) where translationally repressed mRNAs are stored when cells encounter environmental stress. Cytoplasmic polyadenylation element-binding protein (CPEB)4 is a sequence-specific RNA-binding protein and translational regulator. In keeping with the results obtained from the study of other RNA-binding proteins, we found CPEB4 localized in SGs in various arsenite-treated cells. In this study, we identified that Vinexin, a CPEB4-interacting protein, is a novel component of SGs. Vinexin is a SH3-domain-containing adaptor protein and affects cell migration through its association with Vinculin to localize at focal adhesions (FAs). Unexpectedly, Vinexin is translocated from FAs to SGs under arsenite-induced stress. The recruitment of Vinexin to SGs depends on its interaction with CPEB4 and influences SG formation and cell survival. Arsenite-activated c-Jun N-terminal kinase (JNK) signaling enhances the association between CPEB4 and Vinexin, which consequently facilitates SG localization of Vinexin. Taken together, this study uncovers a novel interaction between a translational regulator and an adaptor protein to influence SG assembly and cell survival.

## Introduction

Cytoplasmic mRNAs are dynamically associated with a variety of RNA-binding proteins to control their subcellular localization, stability and translation. These processes are often interconnected and occur in compartmentalized RNA foci. Stress granules (SGs) are one type of cytoplasmic RNA-containing particles, and they form when cells face adverse environment-caused translational silencing. Hypoxia, heat shock and oxidative stress all lead to global translational arrest through phosphorylation-inactivated eukaryotic initiation factor (eIF)2α. As a result, the stalled initiation complex including 40S ribosomal subunits, eIF2α and many other eIFs, as well as mRNAs and RNA-binding proteins, aggregate and accumulate in SGs [1], [2]. SGs are dynamic and reversible structures that allow cells to preserve energy and only make essential proteins under stress and to reinitiate protein synthesis once recovery from the crisis occurs. The formation of SGs is thought to promote survival of stressed cells by decreasing reactive oxygen species and blocking pro-apoptotic signaling [3], [4], [5], [6]. Nevertheless, several RNA-binding proteins involved in neurodegeneration, such as Fused in sarcoma (FUS), TAR DNA-binding protein of 43 kDa (TDP-43) and Ataxin 2, are also the components of SGs. Accumulation of these proteins in SGs was controversially reported to promote or slow down irreversible aggregation of these proteins [7], [8], [9].

The CPEB family of RNA-binding proteins, named CPEB1 to CPEB4 in vertebrates, regulates translation in various tissues [10], [11], [12], [13], [14], [15], [16]. CPEB2, CPEB3 and CPEB4 are more closely related to each other than they are to CPEB1. CPEBs2-4 have 96% and ∼25–35% identity in the C-terminal RNA-binding domain (RBD) and the N-terminal regulatory region, respectively, but they share 45% identity with CPEB1 only in the RBD region [17], [18]. CPEBs shuttle between nucleocytoplasmic compartments with longer retention in the cytoplasm where they regulate target RNA translation [19], [20], [21], [22]. Among them, only CPEB1 was reported to localize in SGs [23]. In this study, we detected SG accumulation of CPEBs2-4 in arsenite-treated cells. The yeast two-hybrid (Y2H) screening and co-immunoprecipitation (co-IP) assay revealed that CPEB4 interacts with an adaptor protein, Vinexin. Vinexin was also identified in our previous Y2H screens using the N-termini of CPEB2 and CPEB3 as the baits [10], [22], indicating that Vinexin likely influences a common function in which all CPEBs2-4, are involved. Three Vinexin isoforms, α, β and γ, encoded from alternatively spliced transcripts, contain a common C-terminal sequence with three Src-homology (SH3) motifs. All Vinexin isoforms bind to Vinculin through their first two SH3 motifs to localize at FAs and promote cell adhesion and spreading [24]. The shortest form, Vinexin β, is most widely expressed in various tissues and cells. In contrast, the expression of Vinexin α and γ is more restricted to skeletal muscle, heart and gonads [24], [25], [26]. Additionally, Vinexin β is present in the nucleus where it interacts with retinoic acid receptor (RAR)γ to negatively control RARγ’s transcriptional activity [27]. Although CPEBs2-4 bind to the SH3 domains of Vinexins and possibly interact with the α, β and γ forms *in* *vivo*, we used the most widely distributed CPEB4 and Vinexin β to characterize the functional significance of their interaction. Up-expression of Vinexin β did not induce FA localization and nuclear retention of CPEB4. Unexpectedly, we found that Vinexin accumulated in SGs not only under the CPEB4-overexpressing condition but also under arsenite and heat shock-induced stress. Further investigation using CPEB double knockout (dKO) mouse embryonic fibroblasts (MEFs) and various truncated Vinexin and CPEB4 mutants confirmed that the recruitment of Vinexin to SGs requires the first two SH3 domains of Vinexin to interact with the proline-rich domains (PRDs) of CPEB4. Moreover, CPEB4-Vinexin interaction is potentiated in response to arsenite-activated JNK signaling that is concomitant with the increased dissociation of Vinexin from Vinculin at FAs. In the CPEB dKO MEFs where Vinexin is unable to localize in SGs or Vinexin knockdown (KD) cells, arsenite-induced SG formation and assembly are significantly impaired, this is accompanied by increased cell death. Therefore, Vinexin is a novel SG component recruited by CPEBs2-4 and plays an active role in promoting SG assembly and cell survival under stress.

## Materials and Methods

### Antibodies and Chemicals

Antibodies used in the study are β-actin (AC-15), flag epitope (F1804), myc (M4439), PABP (P6246) and Vinculin (V9264) from Sigma-Aldrich; TIA-1 (sc-1751) and p-c-Jun (Ser63/73, sc-16312) from Santa Cruz Biotechnology; RFP antibody (GTX59862) and Vinexin (GTX115362) from GeneTex; GFP (#29779) from AnaSpec; c-Jun (#9165) from Cell Signaling Technology and AlexaFluor 488, 594 or 647-conjugated secondary antibodies from Invitrogen. The CPEB4 monoclonal antibody was raised using the N-terminal 458 amino acids (a.a.) of rat CPEB4 (rCPEB4) produced in *Escherichia coli*. The CPEB2 antibody has been described elsewhere [10]. All chemicals were obtained from Sigma-Aldrich.

### Plasmid Construction and Yeast Two-Hybrid Screen

The shRNA clone, TRCN0000123148 (CGGAACGTTCCCTGGAAATTA), targeted against human Vinexin was purchased from the RNAi Core Facility (Academia Sinica). The rat CPEB4 DNA fragment was amplified from cultured neuron cDNAs and cloned into the pcDNA3.1-myc or mCherry red fluorescent protein (RFP) plasmid. The TIA-1 DNA fragment was amplified from human brain cDNA library and cloned into the RFP plasmid. The Vinexin α, β and γ DNA fragments were amplified from mouse testis cDNAs and cloned into the pcDNA3.1-flag or enhanced green fluorescent protein (EGFP) plasmid. The CPEB4 and Vinexin β deletion mutants were generated by PCR amplification and QuikChange Site-Directed Mutagenesis Kit (Stratagene) according to the manufacturer’s protocol. The sequences of primers used to generate variant mutants are listed in Table S1. The mutants, like Δ(SH3)_1–2_ and ΔPRD, of which multiple deletions were sequentially generated. For example, the Δ(SH3)_1–2_ mutant was created using the Δ(SH3)_1_ plasmid as the template to introduce (SH3)_2_ deletion by site-directed mutagenesis. The N-terminus of CPEB4 (a.a. 1–458) was cloned into pGBKT7 and the full length of Vinexin β was cloned into the pGADT7 for yeast two-hybrid screening and interaction following the procedures described previously [22].

### Chemical Treatment

The concentration of arsenite (NaAsO2) used in the study is 0.5 mM except for the Vinexin KD experiment which is 0.1 mM. The other reagents are as follows: 50 µg/ml CHX, 50 µM SP600125, 25 µM PD98059 and 25 µM SB2035800.

### Ethics Statement

The mice used for this study have been approved by Institutional Animal Care and Use Committee (IACUC) of Academia Sinica (protocol number: 12-03-338) and compliant with Taiwan National Science Council guidelines for ethical treatment of animals. All of the experimental protocols were performed in accordance with the guidelines of the IACUC. All efforts were made to minimize the number of animals used and their suffering. The pregnant mice were sacrificed using carbon dioxide euthanasia prior to embryo collection for MEF culture.

### Cell Culture, Lentiviral Infection and Transfection

HEK-293T (ATCC, CRL-11268), HeLa (ATCC, CCL-2), U2OS and COS-7 (ATCC, CRL-1651) cells were cultured in DMEM with 10% fetal bovine serum (FBS). MEFs were isolated from embryonic day 14.5 (E14.5) embryos following the published protocol [28], and cultured in DMEM with 10% FBS, 1X non-essential amino-acid, 200 µM glutamine and 5 µM β-mercaptoethanol. The MEF cells used for this study were in passage 3 to 4. To accomplish genotyping within 2 h for MEF cultures, we routinely determined the genotypes of the CPEB2 and CPEB4 mutant embryos by PCR using tail biopsies and the KAPA mouse genotyping kit (KK7302, KAPA Biosystems) according to the manufacturer’s protocol. The sense primer, CP2F1, 5′-CAAATACTAGCAATTCCCAGGTCC-3′ and two antisense primers CP2R1, 5′-TCTGATGCTACCCATAGGTGGATC-3′ and CP2R2, 5′-TCTGAGCCAAGGAGGAGTTCTG-3′ at a 2∶1∶1 ratio were used to amplify the WT and KO alleles of *cpeb2* gene, respectively. The primers used for CPEB4 and PCR condition have been described previously [29]. For the Vinexin knockdown and rescue experiments, HeLa cells were infected with the lentivirus in the presence of 1 µg/ml polybrene overnight (day 1), replaced with new medium for one day (day 2), selected with 1 µg/ml puromycin for another day (day 3) and then subcultured in 6 cm dishes (day 4). On day 5, the 6-cm plates of knockdown cells were co-transfected with plasmids expressing RFP-Histone 2B (H2B) along with wt or mutant Vinexin using TurboFect tranfection reagent (Fermentas) according to the manufacturer’s protocol. One hour after transfection, the HeLa cells were first labeled with 2 µM of carboxyfluorescein diacetate, succinimidyl ester (CFSE, Invitrogen) in pre-warm phosphate-buffered saline (PBS) for 15 min and then trypsinized and seeded on a 96-well plate of optically clear bottom (CellCarrier 6005550, PerkinElmer) at a density of 40,000 cells/well. The rest of the cells were plated in 3.5-cm dishes and harvested on the next day for western blotting. On day 6, the cells in the 96-well plate were treated or not treated with 0.1 mM arsenite for the indicated time and then processed for high-content imaging analyses.

### Immunostaining and High-Content Imaging for SG Quantification

After the arsenite treatment, cells were washed twice with PBS, fixed with 4% formaldehyde for 20 min, permeabilized with 0.1% Triton X-100 for 5 min, blocked with 3% bovine serum albumin (BSA) for 30 min and then immunostained with TIA-1 antibody for 3 h, followed by 1-h incubation of AlexaFluor 647-conjugated anti-goat IgG and 1 µg/ml of 4′,6-diamidino-2-phenylindole (DAPI). All solutions were prepared or diluted in PBS and antibodies were diluted in 3% BSA in PBS. The entire procedure was carried out at room temperature, and the cells were washed with PBS three times after every change of solution. The 96-well plate was imaged on the ImageXpress Micro Imaging XL System (Molecular Devices) using 40X Apochromat objective lens. Images of each well were acquired in 9 or 25 selected fields and analyzed using the combined modules of MetaXpress ‘Cell Scoring’ and ‘Granularity’ applications to detect the size and the number of punctate structures (i.e., SGs) in each cell. The nuclei were counted based on the DAPI signal and ranged between 8–30 µm in diameter. The cytoplasmic area was contoured according to the CFSE fluorescence with the threshold set at 10–50 µm in diameter and the total area above 700 µm^2^. SGs were scored by counting cytoplasmic TIA-1-immunofluorescent puncta in the range of 1.2 to 3 µm in diameter. For the rescue experiment, the transfected cells were identified by the co-expression of RFP-H2B signal in the nuclei.

### Apoptosis and Cell Viability Measurement

The MEF cells were treated with arsenite for 1 h, washed twice with medium and recovered in culture medium for 4 h or 10 h prior to terminal deoxynucleotidyl transferase dUTP nick end labeling (TUNEL, Roche Applied Science) or PrestoBlue cell viability assay (Invitrogen), respectively, according to the manufacturers’ protocols. Briefly, for the TUNEL assay, cells were fixed with 4% formaldehyde for 1 h at room temperature and then permeabilized with 0.1% Triton X-100 in 0.1% sodium citrate for 2 min on ice, followed by the incubation of TMR red-dUTP/TdT enzyme mixture for 1 h at 37°C and 5 min of DAPI staining at room temperature. For the viability assay, the MEFs and HeLa cells (control and Vinexin knockdown cells) after ± arsenite treatment were harvested at the indicated time and then incubated with the PrestoBlue reagent for 1 h at 37°C, followed by the measurement of absorbance at 585 nm. The cell viability was calculated by dividing the OD_585_ reading from arsenite-treated cells by that from non-treated control.

### Confocal and FRET Image Acquisition and Quantification

MEF, HeLa, U2OS and COS-7 cells grown on the coverslips were treated with arsenite along with or without other indicated reagents for 30 min, and the cells were processed for immunostaining following the aforementioned procedure used for the 96-well plate, except that the incubation with the designated primary antibodies was at 4°C overnight and the stained coverslips were finally mounted with ProLong Gold Antifade reagent (Invitrogen). To quantify fluorescence intensities of designated targets in FAs and SGs, images from ten immunostained cells in each treatment condition were taken under the same exposure setting that maximized the dynamic range of pixel intensity and then analyzed using MetaMorph software. Because the fluorescence signals in SGs and FAs are not saturated (12 bit image, maximal pixel intensity smaller than 4095), they consequently reflect the relative amount of designated proteins present in FAs and SGs. Ten FAs or SGs were randomly selected from each cell image based on the staining of marker, Vinculin or TIA-1, respectively. For live-cell recording, the coverslip of transfected HeLa cells expressing EGFP-Vinexin β and RFP-TIA-1 was transferred to a homemade culture chamber and equilibrated in the recording chamber that contained 5% CO_2_ and was maintained at 37°C for at least 30 min. Time-lapse live images were acquired every 5 min for 30 min after the addition of arsenite. Förster resonance energy transfer (FRET) imaging was conducted using EGFP and mCherry RFP as donor and acceptor, respectively. Emitted light was collected over a spectrum of wavelengths between 490 and 555 nm with band widths of 10.7 nm after excitation of the EGFP donor by the 488 nm laser. Acceptor bleach FRET was performed by continuous bleaching with the 555-nm laser (100% power, 50 iterations) in a chosen region of interest (ROI, circle of 5 µm in diameter) to destroy the acceptor RFP and hence de-quench the donor EGFP. Ten or twenty images were acquired continuously for live or fixed cells, and the acceptor photobleaching was conducted right after the 4^th^ or 10^th^ image, respectively. To resolve faster kinetics for time-lapse ratiometric FRET measurements, continuous fast scans (∼2.6 s) of smaller fields (40×40 µm) of view were used to monitor FRET changes. The FRET efficiency was calculated by ZEN 2011 software (Carl Zeiss) using the following equation: FRET efficiency %  = 100×(average donor intensity of the ROI in the post-bleach image minus that in the pre-bleach image)/average donor intensity of the ROI in the post-bleach image. Acquisition of fluorescent images (spectral imaging, 8-bit or 12-bit) was performed using LSM700 or LSM780 confocal microscope with a Plan-Apochromat 63X/1.25 NA oil objective lens for fixed or live cells, respectively.

### Co-Immunoprecipitation (Co-IP)

The 6 µg of DNA mixtures containing equal amounts of two or three plasmids were co-transfected in a 6-cm dish of 293T cells using TurboFect reagent. The following procedures were conducted at 4°C or on ice unless specified. The overnight transfected cells were lysed in 1 ml IP buffer (50 mM Tris, pH 7.4, 150 mM NaCl, 0.5 mM EDTA, 0.5% NP40, 20% glycerol, 1 mM DTT, 1X protease inhibitor cocktail and 100 µg/ml RNaseA) and centrifuged at 10,000×*g* for 15 min. For co-IP of endogenous CPEB4, a 10-cm plate of 293T cells overexpressing EGFP-Vinexin β or EGFP were mildly crosslinked with 0.8% formaldehyde for 10 min and quenched by the addition of 0.3 M glycine for 5 min at room temperature and then lysed in 1 ml IP buffer. The lysates were sonicated for 10 s and centrifuged at 10,000×*g* for 15 min to obtain the supernatant. The collected supernatants were incubated with protein G beads bound with the indicated IP antibody for 4 h. The beads were washed three times with 1 ml IP buffer and the precipitated proteins were analyzed by western blotting.

## Results

### CPEB4 Interacts with Vinexin and Causes SG Localization of Vinexin

**Figure 1:**
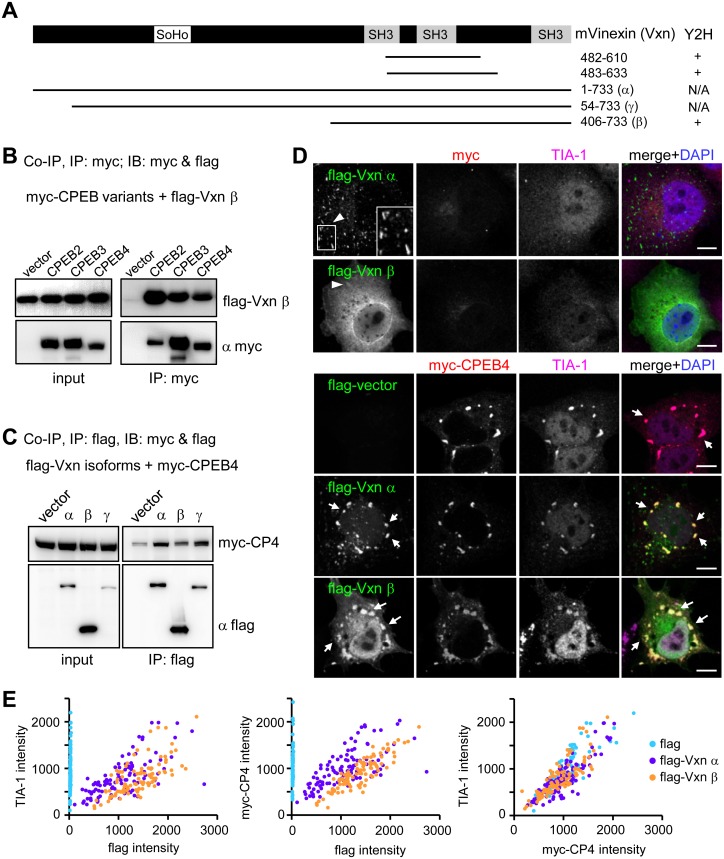
Vinexin interacts with CPEB4 and localizes at SGs in CPEB4-overexpressing cells. (A) Molecular architectures of mouse Vinexin (mVinexin), showing the sorbin homology (SoHo) and three Src-homology (SH3) motifs. The α, β and γ isoforms vary at the N-terminal end. Using the CPEB4 N-terminus as the bait, a yeast two hybrid (Y2H) screen identified two clones corresponding to the a.a. 482–610 and 483–633 of mVinexin α. The full-length mVinexin β was also shown for positive (+) interaction with CPEB4. N/A: not applicable. (B) Co-immunoprecipitation (Co-IP) assay. The 293T cell lysates containing flag-Vinexin β along with myc-tagged CPEB2, 3 or 4 were precipitated with myc IgG and immunoblotted with flag and myc antibodies. IB: immunoblotting, IP: immunoprecipitation. (C) Similarly, the 293T cell lysates containing myc-CPEB4 (myc-CP4) without (vector) or with flag-Vxn α, β or γ, were precipitated with flag IgG and immunoblotted with flag and myc antibodies. (D) Subcellular distribution of flag-Vxn α and β forms in COS-7 cells in the absence or presence of myc-CPEB4 expression. Arrow heads and arrows denote focal adhesion (FA) and stress granule (SG) structures, respectively. TIA-1 immunostained signal detected by the AlexaFluor 647-conjugated secondary antibody was pseudo-colored in magenta and used as a SG marker. The signal intensities of TIA-1, myc-CPEB4, flag-Vxn α and β in one hundred SGs randomly selected from ten transfected cells were pairwisely compared and plotted in (E). Scale: 10 µm.

The yeast two-hybrid screen using the N-terminal 458 amino acids (a.a.) of rat CPEB4 as the bait to probe a mouse brain cDNA library has revealed that a part of the SH3-containing domain of Vinexin (α form, a.a. 482–610 and 483–633) interacted with CPEB4 (Figure 1A). Of note, the clone containing a.a. 483–633 of Vinexin α was also identified in our earlier screens using the N-terminus of CPEB2 or CPEB3 as the bait [10], [22]. Vinexin, also known as Sorbin and SH3 domain-containing protein 3 (SORBS3), is composed of three splice variants, α, β and γ [24], [26]. All of them possess common C-terminal sequences with three SH3 domains (Figure 1A) that bind to diverse signaling molecules and cytoskeletal proteins, such as Sos, Rhotekin, WAVE and Vinculin [24], [30], [31], [32]. Because the N-termini of CPEBs2-4 possess multiple prolin-rich motifs that could potentially interact with the SH3 domains, we first determined whether the shortest β form could bind to all CPEBs. The cell lysates from 293T cells expressing flag-tagged Vinexin β along without or with one of myc-tagged CPEBs2-4 were immunoprecipitated with the myc antibody. Flag-Vinexin β was co-immunoprecipitated with all three myc-CPEBs (Figure 1B). Moreover, myc-CPEB4 was co-precipitated with not only the β form but also α and γ Vinexins (Figure 1C), supporting that the interaction is mediated through the C-terminal SH3 domains of Vinexin. The α and γ isoforms of Vinexin contain an N-terminal sorbin homology (SoHo) domain that interacts with the lipid raft-associated protein, flotillin 1 [33]. The β form in the nucleus negatively regulates the transcriptional activity of RARγ [27]. Nevertheless, all three Vinexins interact with Vinculin to localize at FAs and enhance actin cytoskeletal organization [24], [34]. Because CPEB1 has been found at the leading edge of migrating astrocytes where it regulates local translation of β-catenin RNA [35] and all CPEBs are nucleocytoplasm-shuttling proteins with longer retention time in the cytoplasm [19], [20], [21], [22], [36], we surmised that the interaction with Vinexin could change subcellular distribution of CPEB4. The expression of flag-Vinexin α or β form in COS-7 cells recapitulated the distribution pattern of endogenous Vinexin (Figure 1D). Both forms localized at FA-like structures (indicated by arrow heads) but only the β form was widely distributed in the cytoplasm and nucleus. Using Vinculin immunostaining, we confirmed that these fibrous structures were indeed FAs (Figure S1A). Similar to other RNA-binding proteins, [7], [23], [37], [38], CPEB4, when overexpressed, induced SG structures as indicated by a marker of SGs, T-cell intracytoplasmic antigen (TIA)-1 (Figure 1D). Overexpression of CPEB2 or CPEB3 also induced SG structures as indicated by another SG marker, Poly(A)-binding protein (PABP, Figure S1B). To our surprise, myc-CPEB4 was not recruited to FAs or nucleus by Vinexin α or β; instead, both Vinexin isoforms were localized at SGs in CPEB4-overexressing cells (Figure 1D). Pairwise comparisons of the signal intensities of flag-Vinexin, myc-CPEB4 and TIA-1 in SGs show a high degree of colocalization among these proteins (Figure 1E).

### Arsenite and Heat Shock Stresses Induce Localization of Vinexin in SGs

**Figure 2:**
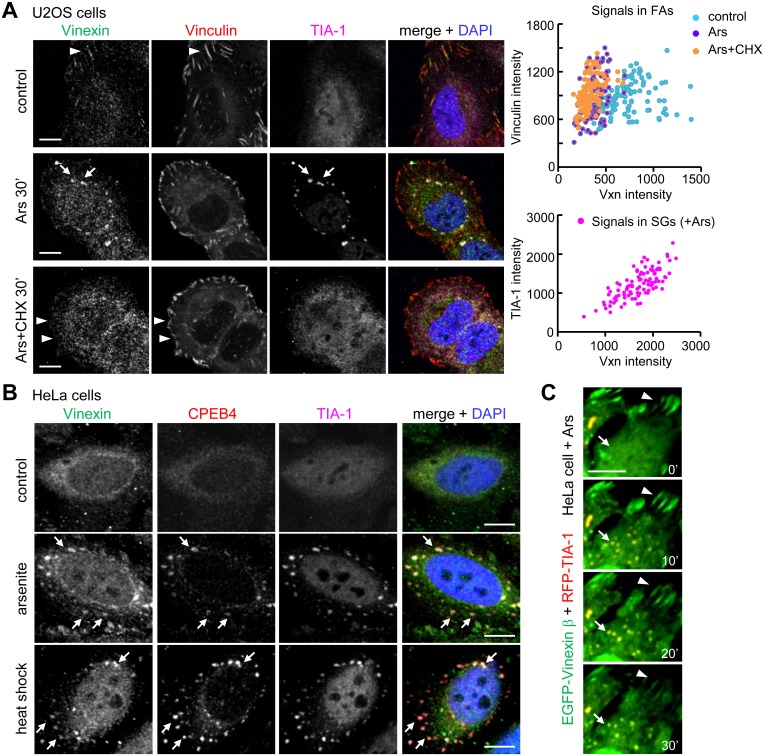
Redistribution of Vinexin from FAs to SGs in arsenite-stressed cells. (A) U2OS cells were treated without (control) or with arsenite ± cycloheximide (CHX) for 30 min prior to immunostaining of Vinexin, Vinculin (FA marker) and TIA-1 (SG marker). Arrow heads and arrows indicate FAs and SGs, respectively. One hundred FAs were randomly selected from ten cell images in each group to quantify the fluorescence intensities of Vinexin and Vinculin. Similarly, a hundred SGs from ten arsenite-treated cells were analyzed for the signals of Vinexin and TIA-1. The top scatter plot shows the reduction of Vinexin signal in FAs after the addition of arsenite. (B) Distribution of Vinexin and CPEB4 in arsenite or heat shock (42°C, 20 min)-treated HeLa cells. Arrows denote colocalization of CPEB4 and Vinexin in SGs. (C) Live imaging of EGFP-Vinexin β and RFP-TIA-1 distribution in HeLa cells treated with arsenite (see Figure S3 for the entire cell images). Arrow heads and arrows indicate FAs and SGs, respectively. Scale: 10 µm.

To follow up on the aforementioned phenomenon, we first evaluated whether endogenous Vinexin is able to cluster at SGs in U2OS and HeLa cells. The affinity-purified Vinexin antibody used here, a gift from Dr. Nagata, was raised against the β form and presumably could detect all three isoforms [32]. Because Vinexin β is the predominant form expressed in various cells [24], [26], the immunostaining pattern of endogenous Vinexin (Figure 2A, 2B) is similar to the distribution of Vinexin β (Figure 1D). The western blots also confirmed that Vinexin β is the major form expressed in HeLa and U2OS cells (Figure S2A). Using Vinculin and TIA-1 as the markers of FAs and SGs, respectively, we detected diffusive Vinexin distribution with some staining signal colocalized with Vinculin at FAs (Figure 2A). After a 30-min incubation with arsenite, the signal intensity of Vinexin decreased in FAs but increased in SGs. Arsenite-induced dissociation of Vinexin from FAs is independent of SG formation because it also occurred when SG assembly was inhibited by cycloheximide (CHX) to block polysome disassembly (Figure 2A). These Vinexin-residing and CHX-sensitive SGs also contain CPEB4 (Figure S2B). Unlike Vinexin, CPEB4 was detected in SGs but not FAs (Figure S2C). In addition to arsenite, heat shock (42°C, 20 min) also triggered SG localization of Vinexin and CPEB4 in HeLa cells (Figure 2B). To monitor the dynamic distribution of Vinexin between FAs and SGs, HeLa cells expressing EGFP-Vinexin β and RFP-TIA-1 were monitored for 30 min after arsenite exposure. Like CPEB4, TIA-1 overexpression also induced SGs in some cells in the absence of arsenite stimulation (Figure S3, the left cell). Colocalization of EGFP-Vinexin β with RFP-TIA-1 in punctate structures occurred as early as 10 min after arsenite incubation, and the disappearance of EGFP-Vinexin β at FAs did not become evident until 20–30 min later (Figure 2C, the entire images are shown in Figure S3). This would indicate that stress initiates the redistribution of subcellular Vinexin, but the source of Vinexin in SGs is not solely translocated from FAs. We also examined whether CPEB4 and Vinexin were present in another cytoplasmic RNA foci, the processing bodies (P-bodies), using mRNA-decapping enzyme (Dcp)1a as the marker. Ectopically expressed CPEB4, but not Vinexin β, was detected in P-bodies, whereas both proteins were preferentially recruited to SGs upon arsenite exposure (Figure S4A). Co-expression of EGFP-Vinexin β along with RFP-CPEB4 in cells treated or not treated with arsenite did not facilitate P-body localization of EGFP-Vinexin β (Figure S4B), indicating differential distribution of Vinexin in the two cytoplasmic RNA foci.

### Translocation of Vinexin to SGs Depends on Its Interaction with CPEB4

**Figure 3:**
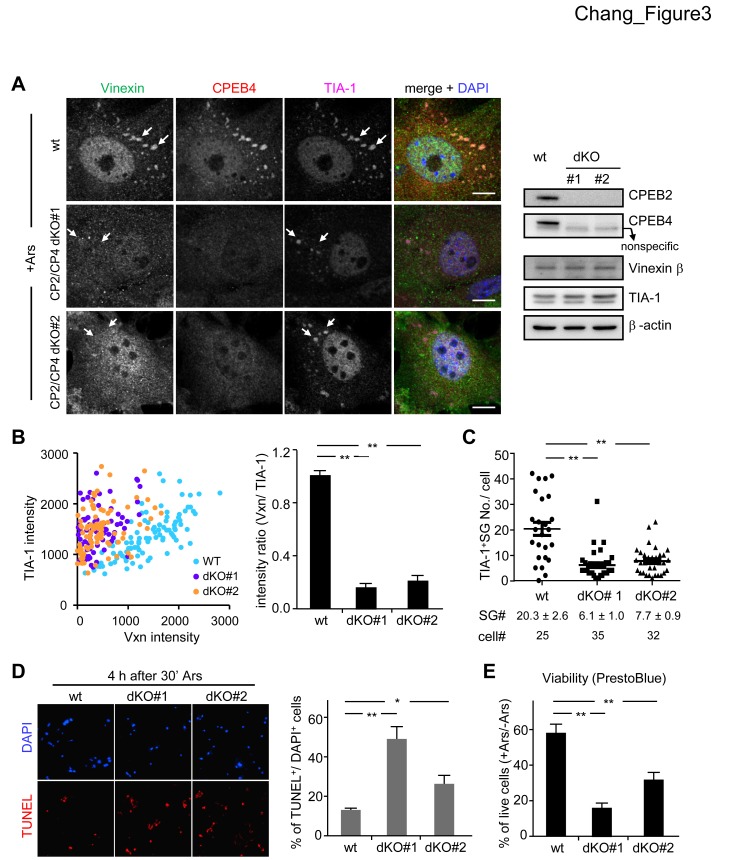
SG localization of Vinexin depends on CPEBs. (A) The wild-type (wt) and CPEB2/CPEB4 double-knockout (CP2/CP4 dKO) MEFs were treated with arsenite and then fixed for immunodetection of Vinexin, CPEB4 and TIA-1. Arrows indicate TIA-1-positive SGs. Scale: 10 µm. The western blots show the expression of indicated proteins in the wt and dKO MEFs. (B) One hundred SGs were randomly selected in ten cell images taken from arsenite-treated wt or dKO MEFs to quantify the signal intensities of Vinexin and TIA-1. The analyzed results show the Vinexin signal is significantly decreased in TIA-1-containing SGs in the dKO cells. (C) For each cell, the number of TIA-1-positive SGs were analyzed and displayed in the dot plot. The average SG number per cell (mean ± s.e.m.) and the number of analyzed cells are listed at the bottom (see Figure S6A for the analyses using CP2KO and CP4 KO MEFs). The percent of (D) apoptotic and (E) survived wt and dKO cells at 4 h and 10 h after arsenite exposure was analyzed by TUNEL and PrestoBlue assays, respectively. The data was presented as mean ± s.e.m. from three independent experiments. One and two asterisks denote *P<0.05 and **P<0.01 (Student’s *t*-test).

Vinexin is recruited to SGs but not P-bodies, both of which contain CPEB4. Thus, it is unclear whether localization of Vinexin in SGs is mediated by its direct association with CPEB4 or is induced under CPEB4-overexpressing stress (Figure 1D) because overexpression of many RNA-binding proteins initiates SG formation in the absence of extracellular stress, such as arsenite. Thus, we compared the distribution of EGFP-Vinexin β under the overexpression of RFP-CPEB4 or RFP-TIA-1 [37]. We found that EGFP-Vinexin β was more accumulated in SGs in CPEB4-overexpressing cells (Figure S5), suggesting that CPEB4 likely recruits Vinexin β to SGs. To demonstrate whether CPEB4 is absolutely required for SG localization of Vinexin, we used MEFs cultured from CPEB4 wild-type (wt) and KO embryos isolated from *cpeb*4+/− heterozygous matings [29] and found normal arsenite-induced SG accumulation of Vinexin in the CPEB4 KO MEFs (Figure S6A). Similarly, CPEB2 KO MEFs also showed normal SG formation in response to arsenite stress (Figure S6A). Since Vinexin interacts with CPEBs 2-4 (Figure 1B) and overexpression of either one of CPEBs2-4 causes SG localization of Vinexin (Figure S6B), the depletion of CPEB4 alone may not be sufficient to abolish SG recruitment of Vinexin. As CPEB2 and CPEB4 are more widely expressed in various tissues and cells than CPEB3 [10], [17], [29], MEFs from two double KO (dKO) embryos isolated from *cpeb2* ^+/−^/*cpeb4* ^+/−^ heterozygous matings were analyzed for Vinexin β distribution in response to arsenite stress. Although the level of Vinexin β was comparable between wt and dKO MEFs in the western blot (Figure 3A), the TIA-1-positive SGs contained evidently decreased Vinexin signal in the arsenite-treated dKO cells (representative images shown in Figure 3A and quantified results shown in Figure 3B). Notably, the average number of TIA-1-containing SGs in the dKO cells decreased significantly (Figure 3C) and this was accompanied with increased apoptosis (Figure 3D) and reduced survival (Figure 3E). These observations reinforce the importance of SG formation in the survival of stressed cells [3], [4], [5], [6].

**Figure 4:**
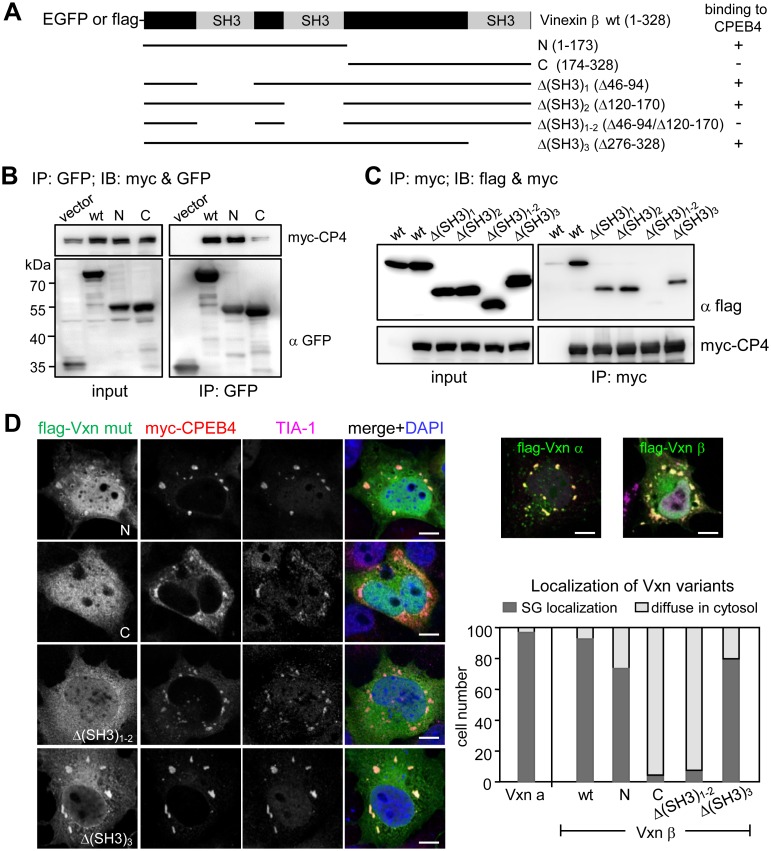
Vinexin binds to CPEB4 and localizes in SGs through its first two SH3 motifs. (A) The domain organization of Vinexin β and various truncated mutants. (B) Co-IP assay. The 293T cells were co-transfected with myc-CPEB4 plasmid along with various EGFP-tagged Vinexin β constructs. The cell lysates were immunoprecipitated with GFP antibody, and immunoblotted with myc and GFP antibodies. (C) The 293T cells expressing myc-CPEB4 and varied SH3-deleted flag-Vinexin β mutants were pulled down with myc IgG and immunodetected with myc and flag antibodies. IP: immunoprecipitation, IB: immunoblotting. (D) SG distribution of wt and Vinexin mutants in COS-7 cells overexpressing myc-CPEB4. If the signal of Vinexin in CPEB4 and TIA-1-positive SGs higher than that in the cytosol was considered as SG-localized (dark grey bar), and the rest was grouped as diffuse in cytosol (light grey bar). One hundred transfected cells per group were analyzed.

If translocation of Vinexin to SGs relied on its association with CPEB4, a Vinexin mutant that is defective in CPEB4-binding should fail to localize in SGs. To map which region of Vinexin is essential for binding to CPEB4, the 293T cells expressing myc-CPEB4 along with varied EGFP-tagged or flag-tagged Vinexin β mutants (Figure 4A) were immunoprecipitated with either a GFP antibody (Figure 4B) or myc antibody (Figure 4C) and immunodetected with GFP, flag and myc antibodies. Co-immunoprecipitations showed that Vinexin interacts with CPEB4 through its first two SH3 domains (Figure 4B, 4C). Moreover, SG localization of these Vinexin mutants correlated with their ability to interact with CPEB4 because the Δ(SH3)_1–2_ and C mutants lacking the CPEB4-binding motif were unable to cluster in SGs (Figure 4D).

### Vinexin Facilitates SG Assembly in a CPEB4-Dependent Manner

**Figure 5:**
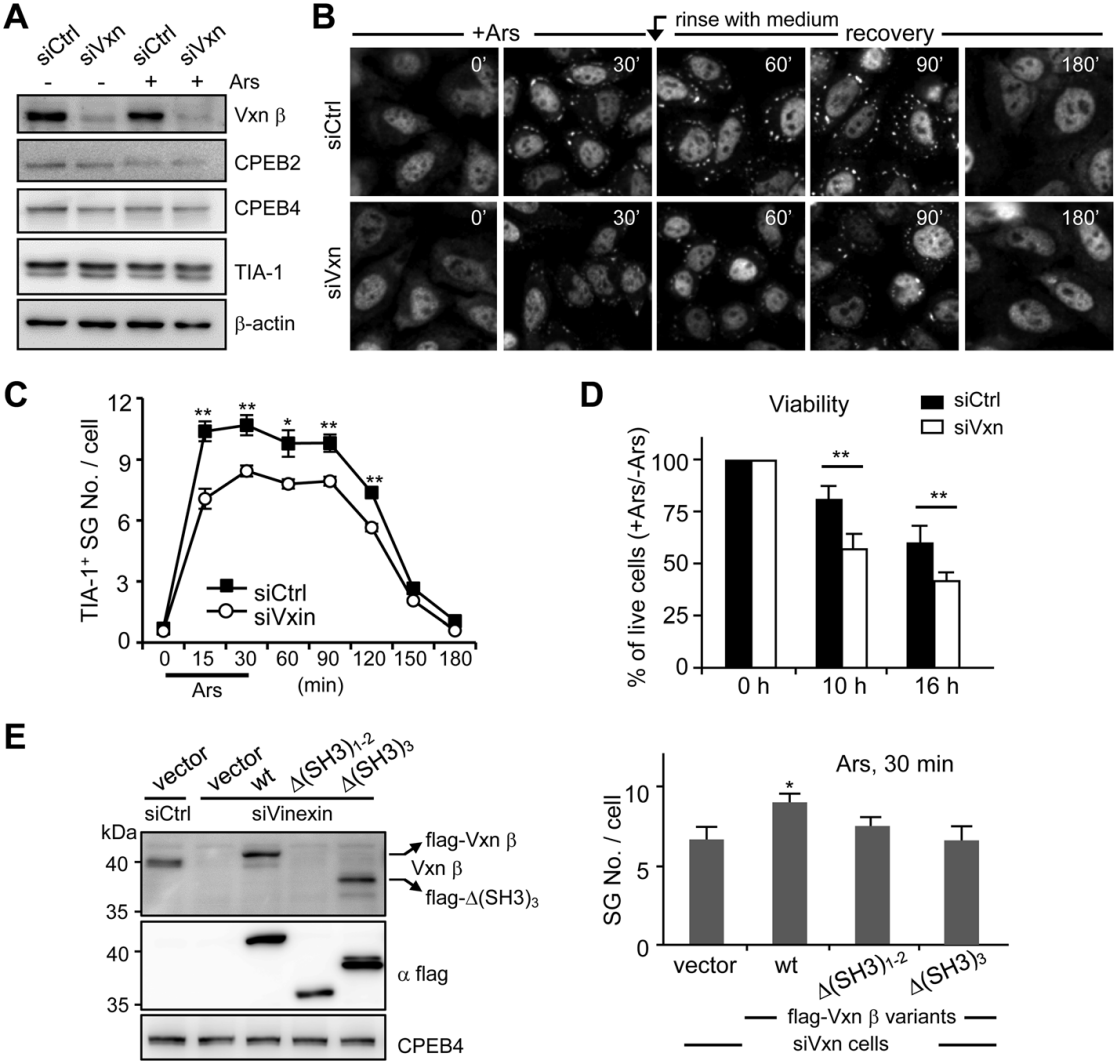
Vinexin promotes SG formation and assembly. HeLa cells infected with lentiviruses expressing without (siCtrl) or with Vinexin shRNA (siVxn) were puromycin-selected, treated with ± arsenite and then harvested at different time points for (A) western blotting or (B) TIA-1 immunostaining to detect SGs. The representative images of siCtrl and siVxn cells during (30 min) and recovering from (180 min) arsenite-induced stress are shown. (C) The number of TIA-1-positive SGs in siCtrl and siVxn cells were analyzed and expressed as the mean ± s.e.m. Approximately 1000 cells in each group collected from three independent experiments were analyzed using the MetaMorph software. The significant difference between siCtrl and siVxn groups at each time point was analyzed with Student’s *t*-test. (D) The percent of survived siCtrl and siVxn cells after arsenite treatment at the indicated time was determined by PrestoBlue viability assay and presented as mean ± s.e.m. from three independent experiments. (E) To test wt or mutant Vinexin in rescuing SG-assembly defect in siVxn cells, the transfected cells were analyzed by western blotting to examine the expression of indicated proteins or TIA-1 immunostaining to score the SG numbers in ∼1000 transfected cells of each group collected from three independent experiments. One and two asterisks denote *P<0.05 and **P<0.01 (Student’s *t*-test).

The impaired SG assembly in the dKO MEFs where Vinexin is incapable to cluster in SGs (Figure 3B) implies that the recruitment of Vinexin to SGs likely plays an active role in promoting SG formation. Thus, we assessed whether SG assembly is defective in Vinexin KD cells. HeLa cells infected with lentiviral particles expressing no (siCtrl) or shRNA targeted against human Vinexin (siVxn) were selected by puromycin to eliminate uninfected cells, these cells were subsequently treated or not treated with arsenite for 30 min. Some cells were harvested for immunoblotting of indicated proteins (Figure 5A), and others were fixed at different time points for TIA-1 immunostaining (Figure 5B, selective images). The analyzed imaging data indicated that the number of TIA-1-positive SGs were significantly reduced, however, the disassembly of SGs after recovering from arsenite stress occurred normally in the siVxn cells (Figure 5C). The survival rate of siVxn cells was significantly decreased as compared with that of siCtrl cells after incubation of arsenite (Figure 5D), supporting the concept that the degree of SG assembly correlates with the survival rate in stressed cells [3], [4], [5], [6]. To rescue the SG assembly defect in the siVxn cells, the knockdown cells were transfected with RFP-H2B along with wt or mutant Vinexin β. The transfected cells were indicated by the presence of nuclear RFP-H2B. Only expression of the wt but not Vinexin β mutants, Δ(SH3)_1–2_ and Δ(SH3)_3_, fully rescued the SG-assembly defect in siVxn cells (Figure 5E). Because the Vinexin antibody from GeneTex was raised against a part of the linker region and the first two SH3 domains (human Vinexin α form, a.a. 354–575), we found this antibody was unable to detect the flag-Δ(SH3)_1-2_ mutant (Figure 5E). Interestingly, the Δ(SH3)_3_ mutant interacted with CPEB4 and localized in SGs (Figure 4) but failed to promote SG assembly, suggesting that the third SH3 motif of Vinexin β recruited additional factors to facilitate SG assembly.

### Arsenite-Activated JNK Signaling Enhances the Interaction of CPEB4 and Vinexin

**Figure 6:**
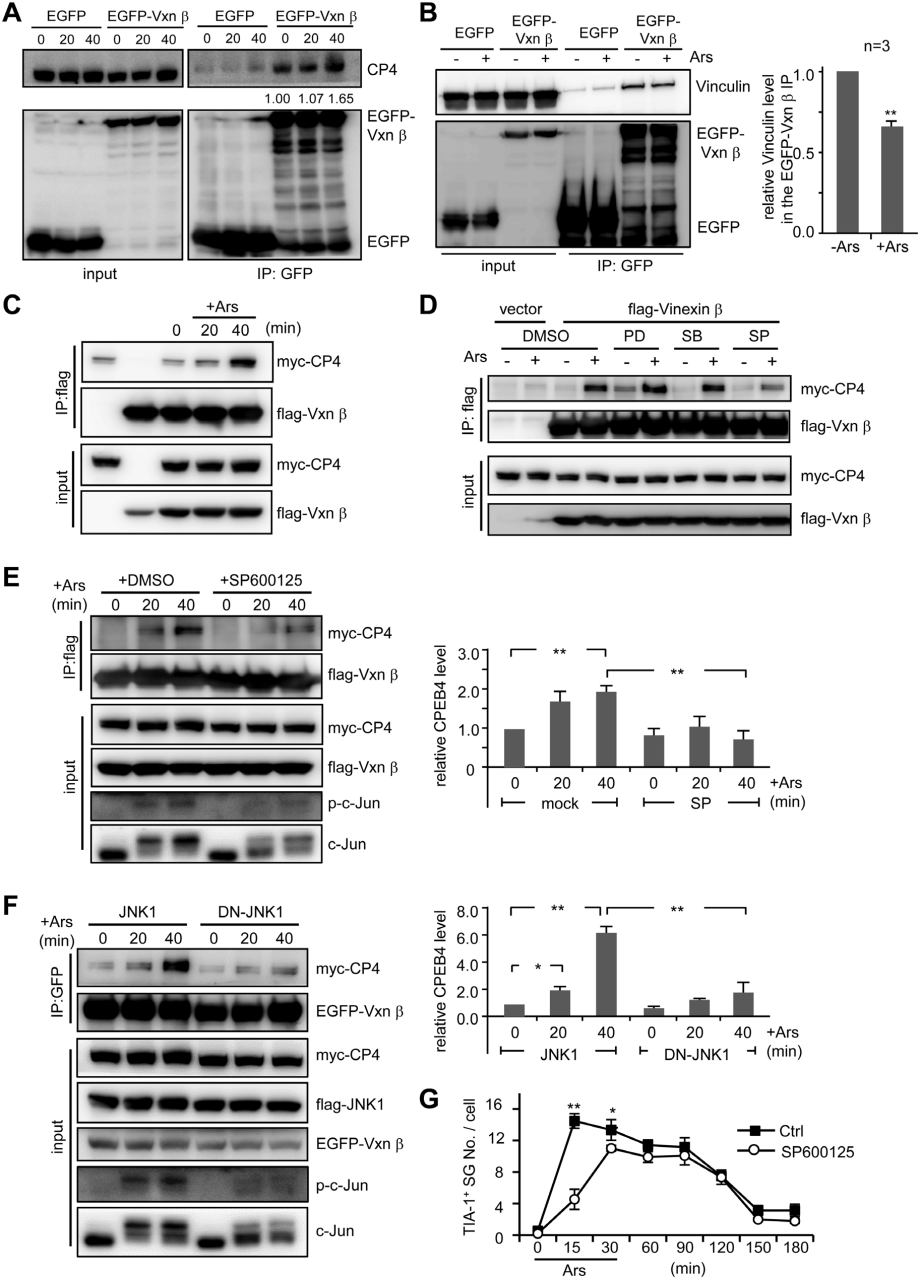
JNK signaling promotes CPEB4-Vinexin interaction in arsenite-treated cells. The 293T cells expressing EGFP or EGFP-Vinexin β were treated with ± arsenite and then harvested for immunoprecipitation using the GFP antibody. The precipitated substances were immunoblotted with (A) CPEB4 and GFP antibodies or (B) Vinculin and GFP antibodies. (C) The 293T cells expressing myc-CPEB4 (CP4) and flag-Vinexin β (Vxn β) were treated with ± arsenite for the denoted times and then precipitated with the flag IgG and immunoblotted using myc and flag antibodies. (D) (E) Similarly to (C) except the cells were pre-treated with various kinase inhibitors, PD98059 (PD), SB2035800 (SB) or SP600125 (SP) for 30 min, followed by arsenite stimulation and co-IP to monitor CPEB4-Vinexin interaction. (F) The 293T cells expressing EGFP-Vinexin β, myc-CPEB4 along with the flag-tagged wt or dominant negative (DN)-JNK1 were harvested at the indicated time after arsenite treatment for co-IP assay. The pull-down substances by the GFP antibody were used for western blotting. For (E, F), the results from three independent experiments were quantified and displayed as mean ± s.e.m. (G) The number of TIA-1-positive SGs in approximately 1000 cells treated with ± SP600125 were analyzed using the MetaMorph software and expressed as the mean ± s.e.m. One and two asterisks denote *P<0.05 and **P<0.01 (Student’s *t*-test).

The first two SH3 domains of Vinexin β are known to interact with cytoskeletal proteins, such as Vinculin and WAVE [24], [31]. Because Vinexin is redistributed from FAs to SGs in arsenite-treated cells (Figure 2A, 2C), we asked whether CPEB4-Vinexin interaction was enhanced under arsenite-activation of mitogen-activated protein kinase (MAPK) pathways, including the extracellular-signal-regulated kinase (ERK), p38 MAPK and JNK [39]. It has been reported that arsenite potently activates both JNK and p38 signaling, but only moderately activates ERK [40]. Using the Vinexin antibody from GeneTex for immunoprecipitation, we detected no CPEB4 in the precipitated substances, likely because of the competitive binding between CPEB4 and the antibody to the first two SH3 domains of Vinexin. To take an alternative approach, the 293T cells expressing EGFP or EGFP-Vinexin β, were treated or not treated with arsenite for the indicated times. The cell lysates were precipitated with GFP antibody and immunoblotted with GFP and CPEB4 antibodies. Using semi-quantitative measurements, the co-IP result showed increasing association of CPEB4 with EGFP-Vinexin β in response to arsenite stimulation (Figure 6A). In contrast, the co-IP experiment showed decreasing interaction between EGFP-Vinexin β and Vinculin in arsenite-treated cells (Figure 6B). To delineate the possible signaling activated by arsenite to regulate Vinexin-CPEB4 interaction, we used cells expressing myc-CPEB4 and flag-Vinexin β for co-IPs. The cell lysates were precipitated with flag IgG, followed by immunodetection with myc and flag antibodies. The ectopically expressed CPEB4 also showed an increased association with Vinexin after the treatment of arsenite (Figure 6C). Nevertheless, this enhanced association was lessened when the cells were treated with the JNK inhibitors (SP600125) but not with the ERK (PD98059) or p38 MAPK (SB2035800) blocker (Figure 6D). More time points were taken for co-IP experiments after arsenite treatment in the presence of JNK inhibitor (Figure 6E) or JNK-dominant negative (DN, the phosphorylation motif Thr_183_Tyr_185_ is mutated to Ala_183_Phe_185_) mutant (Figure 6F). The blockade of JNK activation was confirmed by the reduced phosphorylation of its substrate c-Jun. Quantitative results (bar graphs in Figure 6E, 6F) from three independent co-IP experiments showed that arsenite enhanced CPEB4 and Vinexin interaction in a JNK signaling-dependent manner. Pharmacological inhibition of JNK also impaired SG formation in the arsenite-treated HeLa cells (Figure 6G). Together, these results confirmed that JNK signaling is involved in potentiating CPEB4-Vinexin interaction and SG assembly.

### FRET-Detectable Changes of CPEB4-Vinexin Interaction in SGs

**Figure 7:**
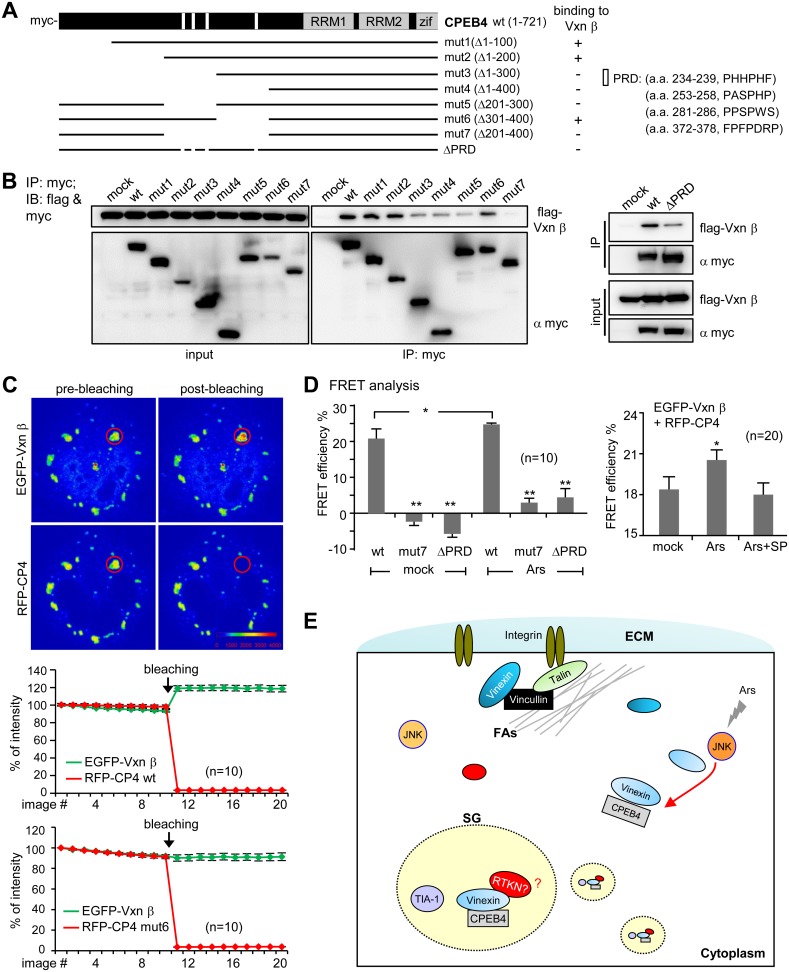
The proline-rich domains (PRDs) of CPEB4 bind to Vinexin in SGs. (A) The domain organization of CPEB4, showing the N-terminal four PRDs and the C-terminal RNA-binding domain composed of two RNA recognition motifs (RRM) and zinc fingers (Zif). The various CPEB4 mutants were illustrated. (B) The 293T lysates containing flag-Vinexin β along with myc-tagged wt or mutant (mut) CPEB4 were precipitated by myc IgG, followed by immunoblotting with myc and flag antibodies. (C) FRET analysis. The plasmids encoding the FRET donor EGFP-Vxn β and acceptor RFP-CPEB4 (CP4) or mutants (CP4mut7 and ΔPRD) were co-transfected to COS-7 cells. The formaldehyde-fixed samples were used for FRET analysis to detect CPEB4-Vineixn interaction in SGs. The example images and the line graph show that the fluorescent signal of EGFP-Vxn β increases after photobleaching the acceptor RFP-CP4 in the selected SG (red circle). In contrast, no increasing change in EGFP signal if RFP-CP4mut7 was used as the acceptor. (D) The changes in fluorescence intensity of EGFP-Vxn β right before and after photobleaching RFP were calculated for FRET efficiency. The FRET interaction between EGFP-Vxn β and RFP-CP4 wt, mut7 or ΔPRD with ± arsenite was determined. Similarly, the FRET interaction of EGFP-Vxn β and RFP-CP4 was also measured in the presence of JNK inhibitor, SP600125 (SP). All of the data were expressed as the mean ± s.e.m. n: the number of SGs in each group (one SG per cell was analyzed by FRET acceptor bleaching). The significant difference between wt and CPEB4 mutants as well as between mock and arsenite (Ars) ± SP treatments was analyzed with Student’s *t*-test. One and two asterisks denote *P<0.05 and **P<0.01. (E) Schematic model of arsenite-induced redistribution of CPEB4 and Vinexin to SGs. Arsenite stress activates JNK signaling, which somehow promotes the association of CPEB4 and Vinexin to recruit Vinexin in SGs. Once translocated to SGs, Vinexin plays an active role in facilitating SG assembly, most likely recruiting additional factors (e.g., RTKN: Rhotekin) through its third SH3 motif. Meanwhile, the dissociation of Vinexin from cytoskeletal proteins, such as Vinculin, weakens focal adhesions and promotes Vinexin translocation from FAs to SGs. ECM, extracellular matrix.

To examine the spatial dynamics of CPEB4-Vinexin association in arsenite-stressed cells, we designed an intermolecular FRET-based reporter system that exploits the physical interaction of CPEB4 and Vinexin in SGs. EGFP and mCherry (RFP) were used as the FRET donor and acceptor, respectively. The COS-7 cells expressing EGFP-Vinexin β along with RFP-CPEB4 or RFP-TIA-1 were monitored live for FRET efficiency (Figure S7A). If the two fluorescent proteins interacted *in* *vivo*, the fluorescence intensity of the donor EGFP will increase upon bleaching the acceptor RFP. The calculated FRET efficiency of EGFP-Vinexin β with RFP-CPEB4 and RFP-TIA-1 in SGs is ∼14.5% and 2.5%, respectively, suggesting that the binding of EGFP-Vinexin β and RFP-CPEB4 in SGs could be specifically detected by FRET (Figure S7A). We then fixed the cells with formaldehyde to prevent the motion of SGs for continuously steady imaging. FRET analysis of these fixed samples also showed a specific interaction between EGFP-Vinexin β and RFP-CPEB4 in SGs (Figure S7B). In order to identify a Vinexin-binding defective mutant of CPEB4, the 293 T cells expressing flag-Vinexin β along with various myc-tagged CPEB4 mutants (Figure 7A) were pulled down with myc IgG and immunodetected with flag and myc antibodies. Because the SH3 motif is known to bind to proline-rich domain (PRD) containing the PXXP sequence (X, any amino acid) [41], we used SH3-Hunter web server [42] to identify four PRDs in CPEB4 and constructed ΔPRD mutant (Figure 7A). Mut3, mut4, mut5, mut7 and ΔPRD were not efficiently co-precipitated with Vinexin β, indicating that the four PRDs located in a.a. 200–400 of CPEB4 are essential for binding to Vinexin (Figure 7B). FRET analysis also confirmed that RFP-tagged mut7 and ΔPRD did not interact with EGFP-Vinexin β in SGs (Figure 7C, 7D). Furthermore, it was FRET-detectable that arsenite-increased Vinexin-CPEB4 interaction in SGs, which was hindered by the presence of JNK inhibitor (Figure 7D). Together, these results buttress the concept that arsenite-activated JNK signaling promotes the association of CPEB4 and Vinexin which facilitates SG assembly (Figure 7E).

## Discussion

Vinexin plays versatile roles in regulating signal transduction, gene expression and cytoskeletal organization through binding to a variety of partners, including signaling molecules like Sos and Rhotekin [30], [32], transcription factor RARγ, and actin-binding proteins, such as WAVE and Vinculin [24], [31], [43]. Vinexin α and γ also interact with lipid raft-localized flotillin through their SoHo domains [33], [44], [45]. In this study, we have identified a novel interaction between the adaptor protein, Vinexin, and the translational regulator, CPEB4 as well as their co-dependent functions in promoting SG assembly. CPEB4 recruits Vinexin to SGs in response to adverse environments such as heat shock or arsenite-induced stress. Moreover, CPEB4 binds to the first two SH3 domains of Vinexin that are also required for interacting with Vinculin. Vinculin is an actin-binding protein, which is localized at FAs and cell-cell adhesion sites. [24], [46]. Thus, acute exposure to high-dose arsenite (0.5 mM, 30 min) not only induces SG formation but also weakens focal adhesions by promoting CPEB4-Vinexin interaction and Vinexin-Vinculin dissociation, respectively. Redistribution of Vinexin from FAs to SGs, in part, contributes to arsenite-induced pleiotropic cellular changes. It has been reported that prolonged exposure to low-dose arsenite (5 µM, 48 h) alters cell migration and focal adhesions via inhibition of the activity of focal adhesion kinase (FAK) and the FAK substrate paxillin [47]. Although we have identified that stress-activated JNK signaling is critical for the enhanced binding between CPEB4 and Vinexin, the molecular changes, possibly through phosphorylation in CPEB4 and/or Vinexin to increase their association, as well as whether FAK and other signaling pathways regulate the dissociation of Vinexin and Vinculin by high-dose arsenite, will require further investigation.

An unresolved issue is the nature of the mRNP complex within SGs. We have identified Vinexin as a novel cofactor of SG assembly and showed that its localization to SGs is dependent on its interaction with CPEBs2-4. The interaction of CPEBs2-4 and Vinexin is mediated through multiple binding of PRDs and SH3 motifs of these proteins, respectively, because several PRDs are also identified in CPEB2 and CPEB3 using the SH3-Hunter server [42]. SG accumulation of Vinexin is not affected unless most of CPEB2-4 proteins are depleted as evidenced in *cpeb2*/*cpeb4* dKO MEFs, indicating that CPEBs2-4 are functionally redundant in recruiting Vinexin to SGs. Since the Δ(SH3)_3_ mutant, interacted with CPEB4 and localized in SGs but failed to rescue the SG assembly defect in the Vinexin knockdown cells, Vinexin in SGs likely serves as a scaffolding adaptor to anchor additional molecules through its third SH3 motif to promote SG formation. One possible candidate is the Ras homolog gene family member (Rho)-effector, Rhotekin [32], since a previous study has identified RhoA and its downstream kinase, ROCK1 are recruited to SGs to facilitate SG formation [48]. Rhotekin binds to the GTP-bound Rho and reduces the GTPase activity of Rho proteins to keep Rho in an active state [49], [50]. We surmise that the third SH3 domain of Vinexin may indirectly assist SG localization of Rho via Rhotekin or other factors to consolidate SG structures (Figure 7E).

Recent studies have uncovered the spectrum of proteins that are unique or commonly shared between SGs and P-bodies. The latter RNA foci are the site of RNA degradation [2], [51]. Several studies showed a transient interaction between SGs and P-bodies during arsenite treatment [23], [52]. This dynamic connection between the two structures likely allows exchanges of mRNPs between them to differentially control mRNA metabolism (i.e., storage or decay) in the cytoplasm. Nevertheless, the dynamic assembly of these compartmentalized mRNPs into higher order structures, such as SGs and P-bodies, in response to extracellular signaling remains partially characterized. Although CPEB4 is detected in P-bodies, we could barely observe Vinexin signal here even under overexpressing condition. Moreover, CPEB4 is mostly accumulated in SGs rather than P-bodies if the two types of RNA granules are simultaneously present in stressed cells. One of the reasons that Vinexin is not recruited by CPEB4 in P-bodies of non-stressed cells is likely due to the competitive binding of Vinexin with the cytoskeletal organizer, Vinculin. The interaction of Vinexin with CPEB4 and Vinculin is coordinately switched, in part, by stress-activated JNK signaling, and co-localization of Vinexin and CPEB4 in SGs occurs in cells facing environmental stress (arsenite or heat shock) or CPEB4-overexpressing proteotoxic stress. Thus, Vinexin is a unique component of SGs and most likely serves as scaffolding proteins for anchoring signaling transduction pathways to the SG and promoting SG organization and assembly.

## Supporting Information

Figure S1 **Distribution of ectopically expressed Vinexin and CPEB in COS-7 cells.** (A) Vinexin α and β were colocalized with Vinculin in focal adhesions (FAs). COS-7 cells transfected with the plasmid expressing flag-tagged α or β Vinexin (Vxn) were immunostained with Vinculin and TIA-1 antibodies to denote FAs and SGs, respectively. TIA-1 immunostained signal detected by the AlexaFluor 647-conjugated secondary antibody is pseudo-colored in magenta. Arrow heads indicate FAs. (B) CPEBs2-4 are localized in SGs. COS-7 cells transfected with the plasmid expressing EGFP or EGFP-tagged CPEB2, CPEB3 or CPEB4 were immunostained with PABP antibody to denote SGs. PABP: poly(A)-binding protein. Scale: 10 µm.
(TIF)

Figure S2 **CPEB4 co-localized with Vinexin in SGs but not FAs in U2OS cells.** (A) Vinexin β is the major form expressed in HeLa and U2OS cells. Vinexin expression was detected in siCtrl, vinexin knockdown (siVinexin), and overexpression (flag-Vxn α, β, γ) HeLa and U2OS cells. (B) (C) U2OS cells were treated without (control) or with arsenite ± cycloheximide (CHX) for 30 min prior to immunostaining of Vinexin, CPEB4, TIA-1 (SG marker) and Vinculin (FA marker). TIA-1 immunostained signal detected by the AlexaFluor 647-conjugated secondary antibody is pseudo-colored in magenta. Arrow heads and arrows indicate FAs and SG, respectively. CPEB4 signal in FAs and SGs in the arsenite-treated cells was quantified and plotted against the fluorescence intensity of Vinculin (red dot) and TIA-1 (magenta dots), respectively. Scale: 10 µm.
(TIF)

Figure S3 **Redistribution of Vinexin from FAs to SGs in arsenite-stressed cells.** Live imaging of EGFP-Vinexin β and RFP-TIA-1 distribution in HeLa cells treated with arsenite. Arrow heads and arrows indicate FAs and SGs, respectively. The selected region of interest (ROI) was shown in higher magnification. Scale: 10 µm.
(TIF)

Figure S4 **Distribution of Vinexin and CPEB4 in SGs and P-bodies.** (A) Distribution of myc-CPEB4 and flag-Vinexin β between SGs and P-bodies in HeLa cells treated with ± arsenite. SGs and P-bodies were indicated by immunostained signals of TIA-1 and Dcp1a, respectively. Dcp1a immunostained signal detected by the AlexaFluor 647-conjugated secondary antibody is pseudo-colored in magenta. (B) Co-expression of EGFP-Vinexin β and RFP-CPEB4 in HeLa cells treated with ± arsenite. Arrow heads and arrows denote P-bodies and SGs, respectively. ROI: region of interest. Scale: 5 µm.
(TIF)

Figure S5 **Accumulation of EGFP-Vinexin β at SGs caused by overexpression of RFP-TIA-1 or RFP-CPEB4 in COS-7 cells.** (A) The expression levels of EGFP-Vinexin β along with RFP, RFP-TIA-1 or RFP-CPEB4 in the transfected COS-7 cells were detected using western blotting with the RFP and GFP antibodies. (B) The signal intensities of EGFP-Vxn β and RFP-TIA-1/or RFP-CPEB4 in one hundred SGs from ten transfected cells were quantified and plotted. Scale: 10 µm.
(TIF)

Figure S6 **Overexpression of either one of CPEBs2-4 induces SG localization of Vinexin.** (A) The wild-type (wt), CPEB2 knockout (CP2KO) and CPEB4 knockout (CP4KO) MEFs were treated with arsenite and then fixed for immunodetection of Vinexin, CPEB4 and TIA-1. Arrows indicate TIA-1-positive SGs. One hundred SGs were randomly selected in ten cell images taken from arsenite-treated wt or KO MEFs to quantify the signal intensities of Vinexin and TIA-1 in SGs. For each cell, the number of TIA-1-positive SGs was analyzed and displayed in the dot plot. The average SG number per cell (mean ± s.e.m.) and the number of analyzed cells are listed at the bottom. (B) COS-7 cells transfected with the plasmid expressing myc-tagged CPEB2, CPEB3 or CPEB4 were immunostained with Vinexin and TIA-1 antibodies. Arrows indicate SGs. The immunostained signal of Vinexin was plotted against that of TIA-1 or myc-CPEB in a hundred SGs randomly selected from ten transfected cells. Scale: 10 µm.
(TIF)

Figure S7 **FRET detection of CPEB4-Vinexin interaction in SGs.** The plasmids encoding the FRET donor EGFP-Vinexin (Vxn) β and acceptor RFP-CPEB4 (CP4) or RFP-TIA-1 were co-transfected to COS-7 cells. (A) The live cells were used for FRET analysis to detect the interaction of Vinexin with CPEB4 or TIA-1 in the selected SG (red circle). The example images show that the fluorescent signal of EGFP-Vxn β increases after photobleaching the acceptor RFP-CP4 but not RFP-TIA-1. The changes in fluorescence intensity of EGFP right before and after bleaching RFP were calculated as FRET efficiency. All of the data were expressed as the mean ± s.e.m. n: the number of SGs and cells in each group (one SG per cell was performed for FRET analysis). (B) Similar to (A), except the fixed samples were used for FRET analysis.
(TIF)

Table S1 **The sequences of primers used for constructing CPEB4 and Vinexin mutants.**
(DOCX)
