# Supplementary figures and images for: Arsenite-Activated JNK Signaling Enhances CPEB4-Vinexin Interaction to Facilitate Stress Granule Assembly and Cell Survival
Source: PLoS One. 2014 Sep 19;9(9):e107961. doi: 10.1371/journal.pone.0107961 (PMC4169592; doi:10.1371/journal.pone.0107961)

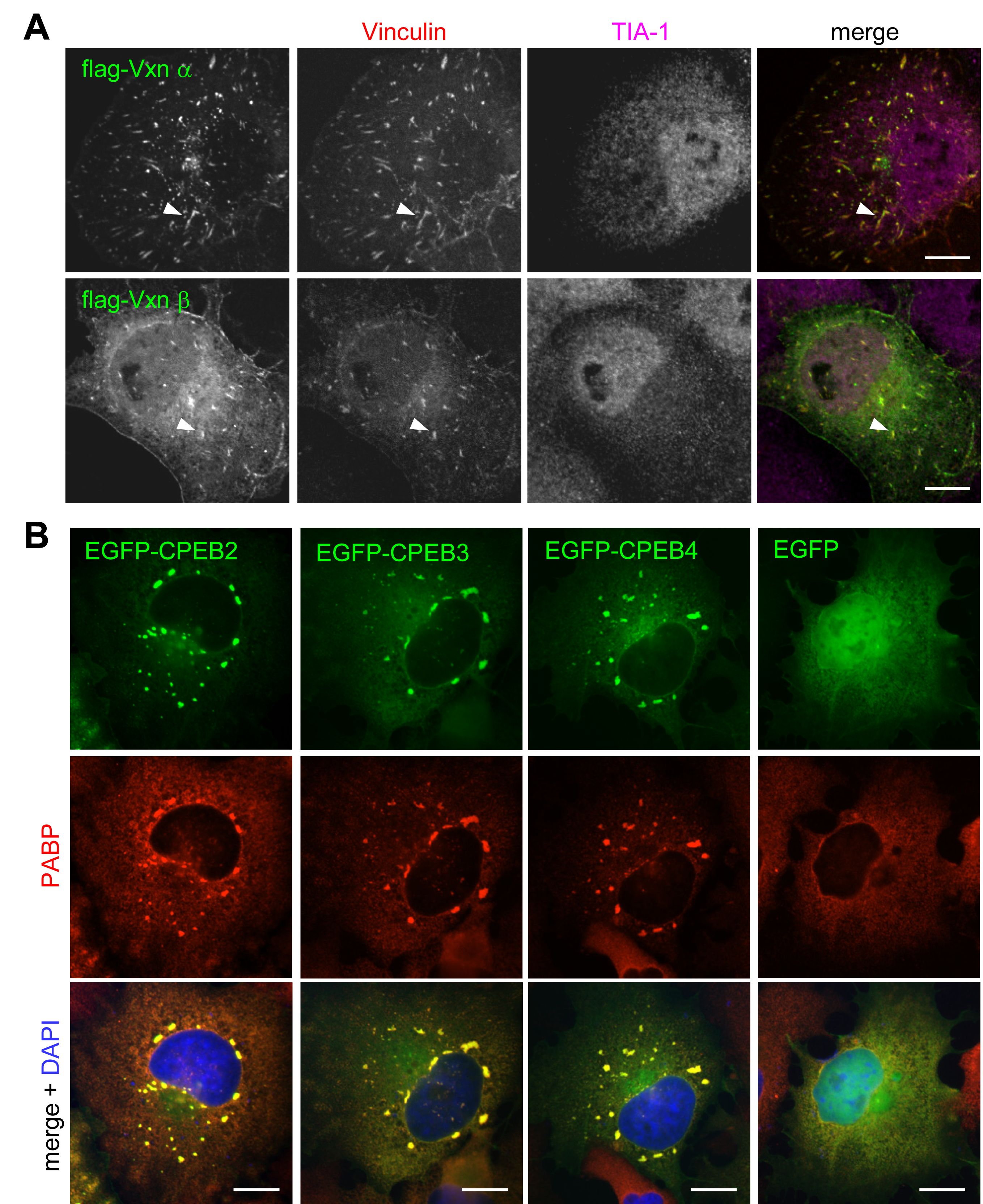

Supplement: Figure S1 — Distribution of ectopically expressed Vinexin and CPEB in COS-7 cells. (A) Vinexin α and β were colocalized with Vinculin in focal adhesions (FAs). COS-7 cells transfected with the plasmid expressing flag-tagged α or β Vinexin (Vxn) were immunostained with Vinculin and TIA-1 antibodies to denote FAs and SGs, respectively. TIA-1 immunostained signal detected by the AlexaFluor 647-conjugated secondary antibody is pseudo-colored in magenta. Arrow heads indicate FAs. (B) CPEBs2-4 are localized in SGs. COS-7 cells transfected with the plasmid expressing EGFP or EGFP-tagged CPEB2, CPEB3 or CPEB4 were immunostained with PABP antibody to denote SGs. PABP: poly(A)-binding protein. Scale: 10 µm. (TIF) [file pone.0107961.s001.tif]

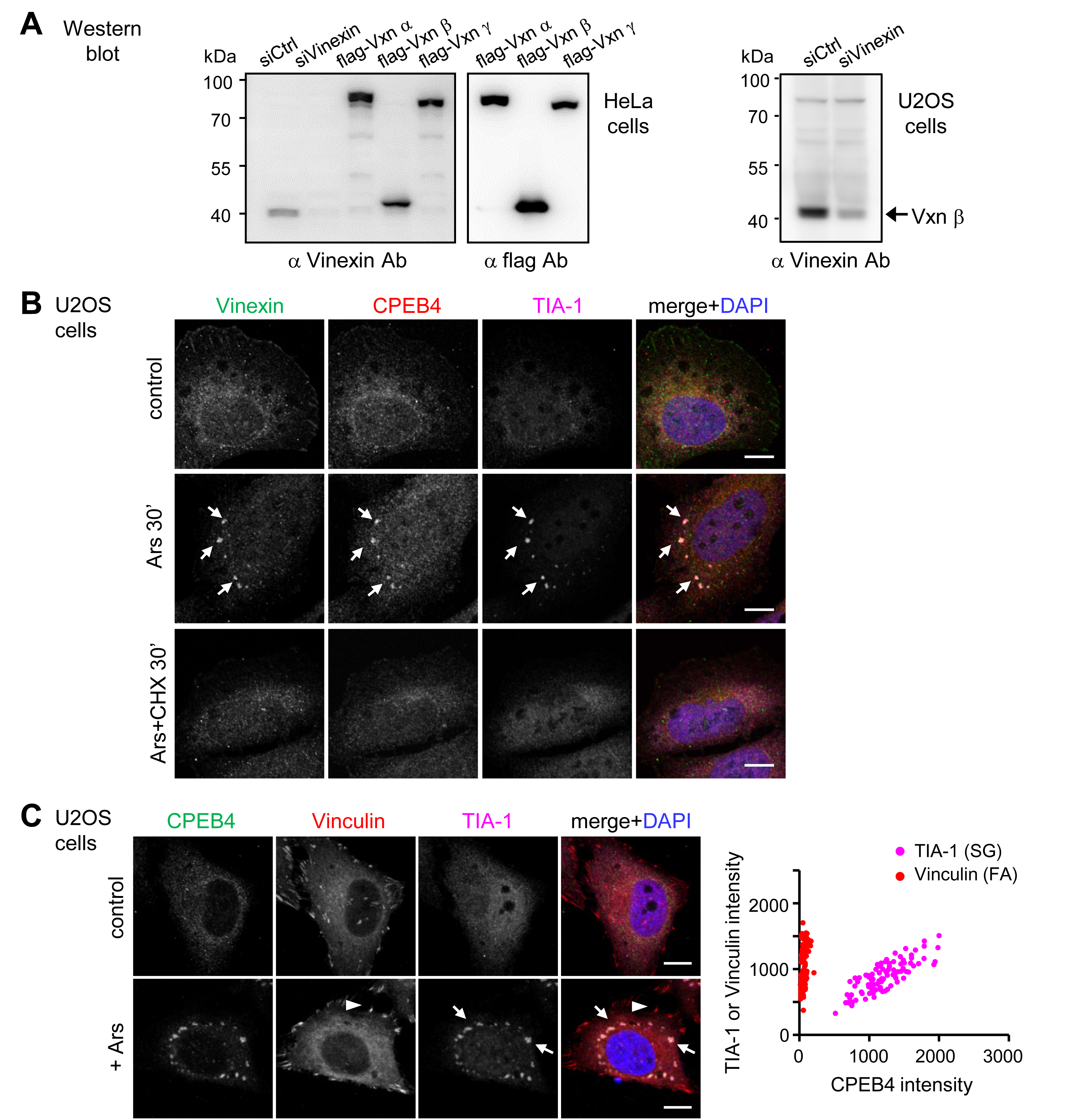

Supplement: Figure S2 — CPEB4 co-localized with Vinexin in SGs but not FAs in U2OS cells. (A) Vinexin β is the major form expressed in HeLa and U2OS cells. Vinexin expression was detected in siCtrl, vinexin knockdown (siVinexin), and overexpression (flag-Vxn α, β, γ) HeLa and U2OS cells. (B) (C) U2OS cells were treated without (control) or with arsenite ± cycloheximide (CHX) for 30 min prior to immunostaining of Vinexin, CPEB4, TIA-1 (SG marker) and Vinculin (FA marker). TIA-1 immunostained signal detected by the AlexaFluor 647-conjugated secondary antibody is pseudo-colored in magenta. Arrow heads and arrows indicate FAs and SG, respectively. CPEB4 signal in FAs and SGs in the arsenite-treated cells was quantified and plotted against the fluorescence intensity of Vinculin (red dot) and TIA-1 (magenta dots), respectively. Scale: 10 µm. (TIF) [file pone.0107961.s002.tif]

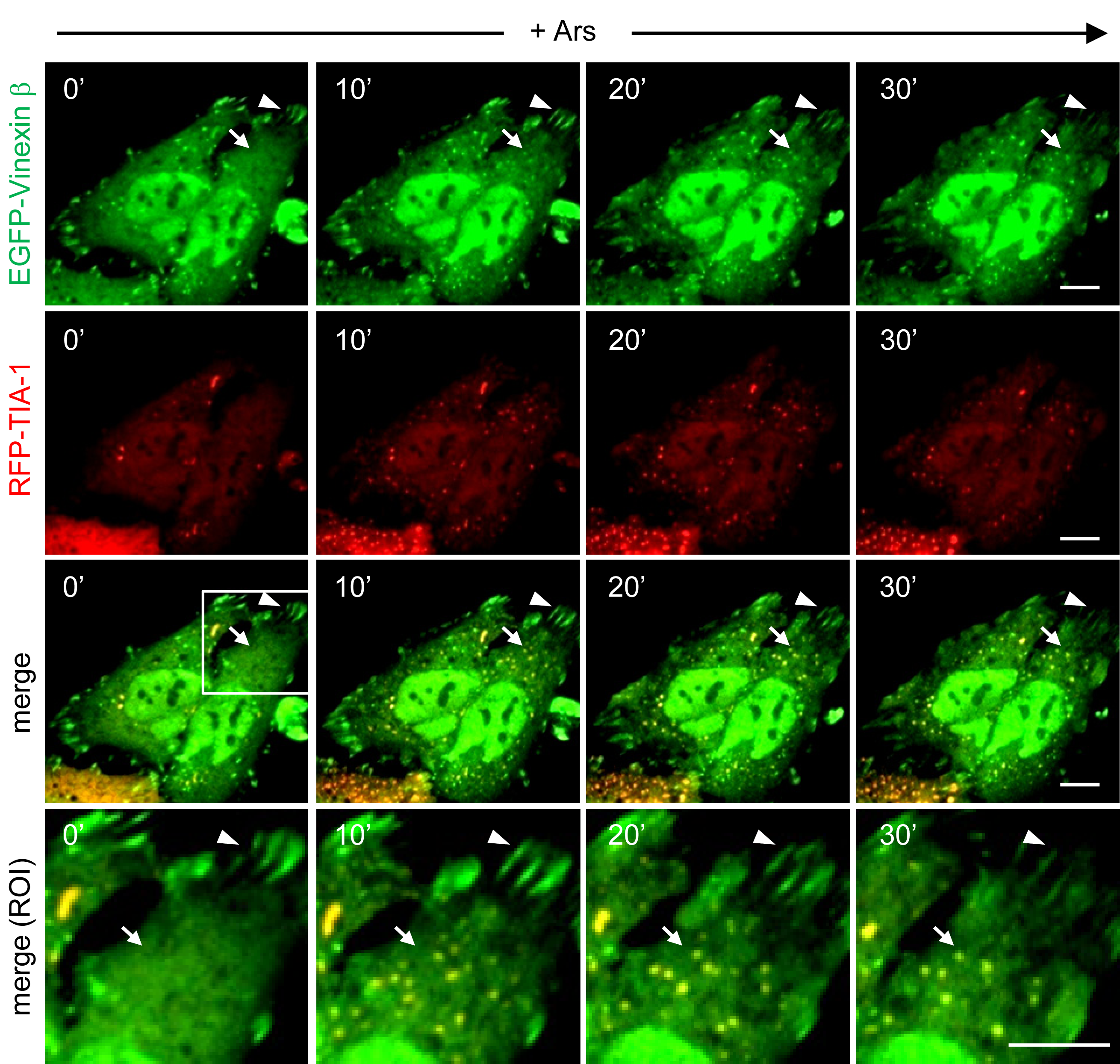

Supplement: Figure S3 — Redistribution of Vinexin from FAs to SGs in arsenite-stressed cells. Live imaging of EGFP-Vinexin β and RFP-TIA-1 distribution in HeLa cells treated with arsenite. Arrow heads and arrows indicate FAs and SGs, respectively. The selected region of interest (ROI) was shown in higher magnification. Scale: 10 µm. (TIF) [file pone.0107961.s003.tif]

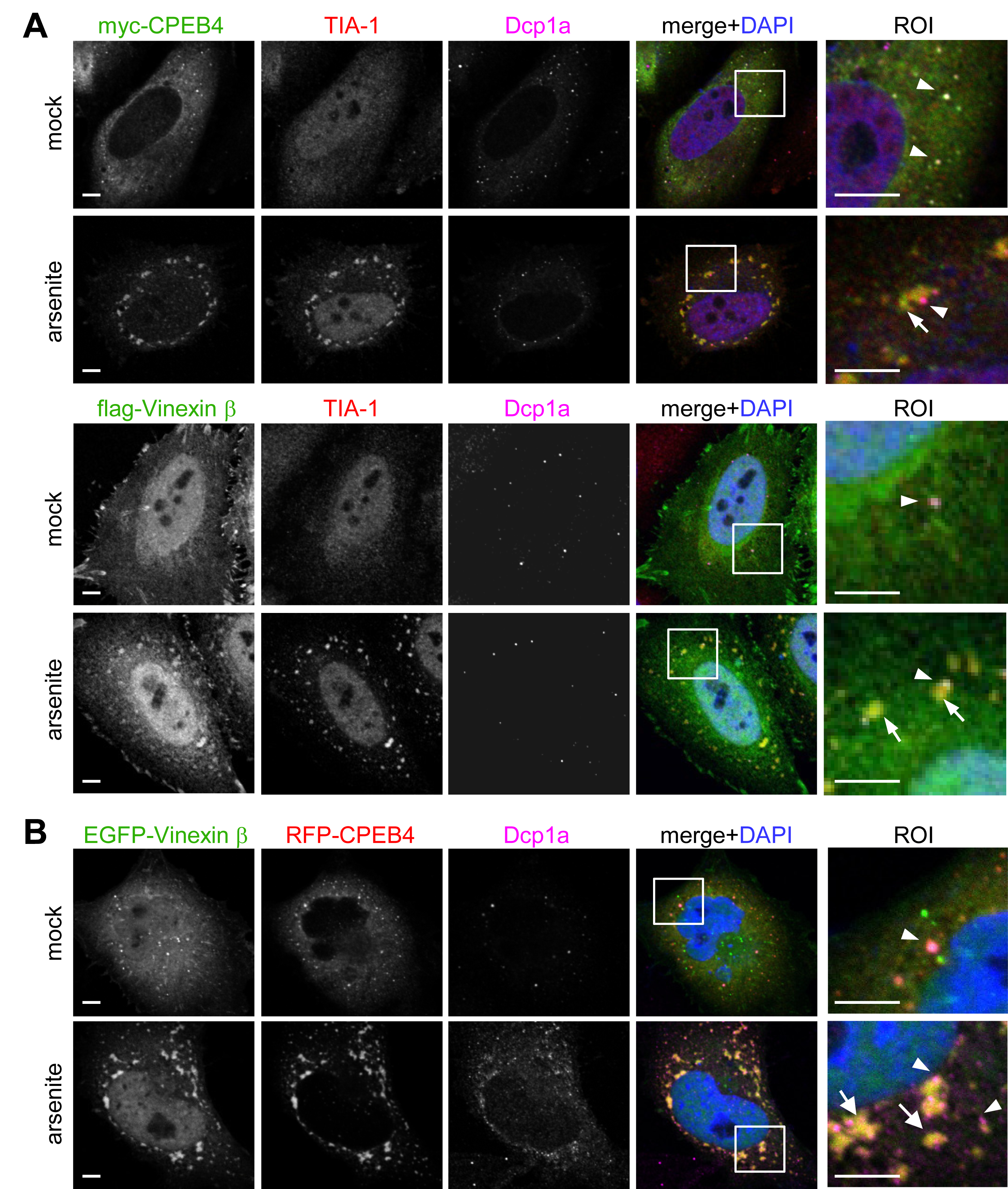

Supplement: Figure S4 — Distribution of Vinexin and CPEB4 in SGs and P-bodies. (A) Distribution of myc-CPEB4 and flag-Vinexin β between SGs and P-bodies in HeLa cells treated with ± arsenite. SGs and P-bodies were indicated by immunostained signals of TIA-1 and Dcp1a, respectively. Dcp1a immunostained signal detected by the AlexaFluor 647-conjugated secondary antibody is pseudo-colored in magenta. (B) Co-expression of EGFP-Vinexin β and RFP-CPEB4 in HeLa cells treated with ± arsenite. Arrow heads and arrows denote P-bodies and SGs, respectively. ROI: region of interest. Scale: 5 µm. (TIF) [file pone.0107961.s004.tif]

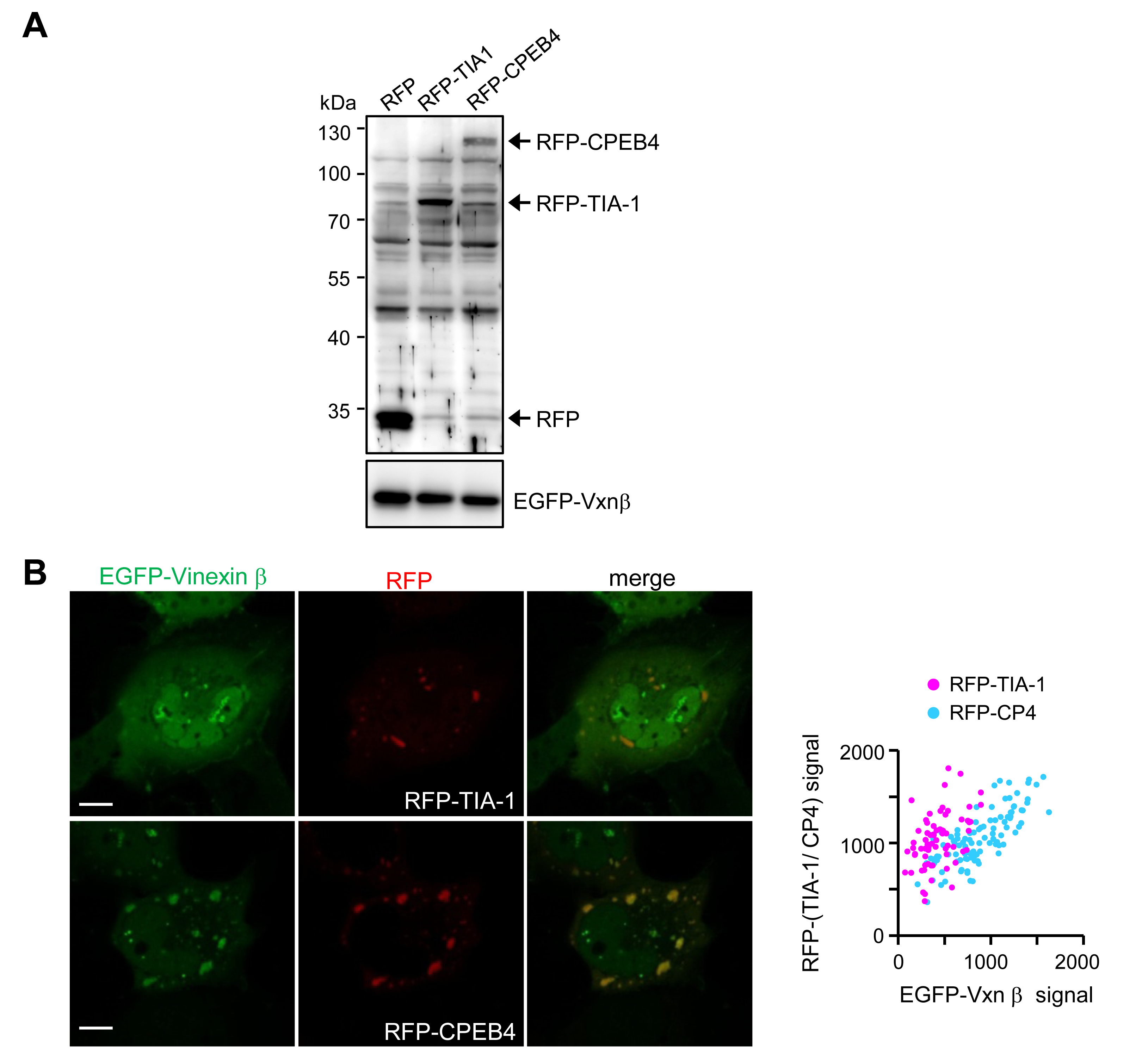

Supplement: Figure S5 — Accumulation of EGFP-Vinexin β at SGs caused by overexpression of RFP-TIA-1 or RFP-CPEB4 in COS-7 cells. (A) The expression levels of EGFP-Vinexin β along with RFP, RFP-TIA-1 or RFP-CPEB4 in the transfected COS-7 cells were detected using western blotting with the RFP and GFP antibodies. (B) The signal intensities of EGFP-Vxn β and RFP-TIA-1/or RFP-CPEB4 in one hundred SGs from ten transfected cells were quantified and plotted. Scale: 10 µm. (TIF) [file pone.0107961.s005.tif]

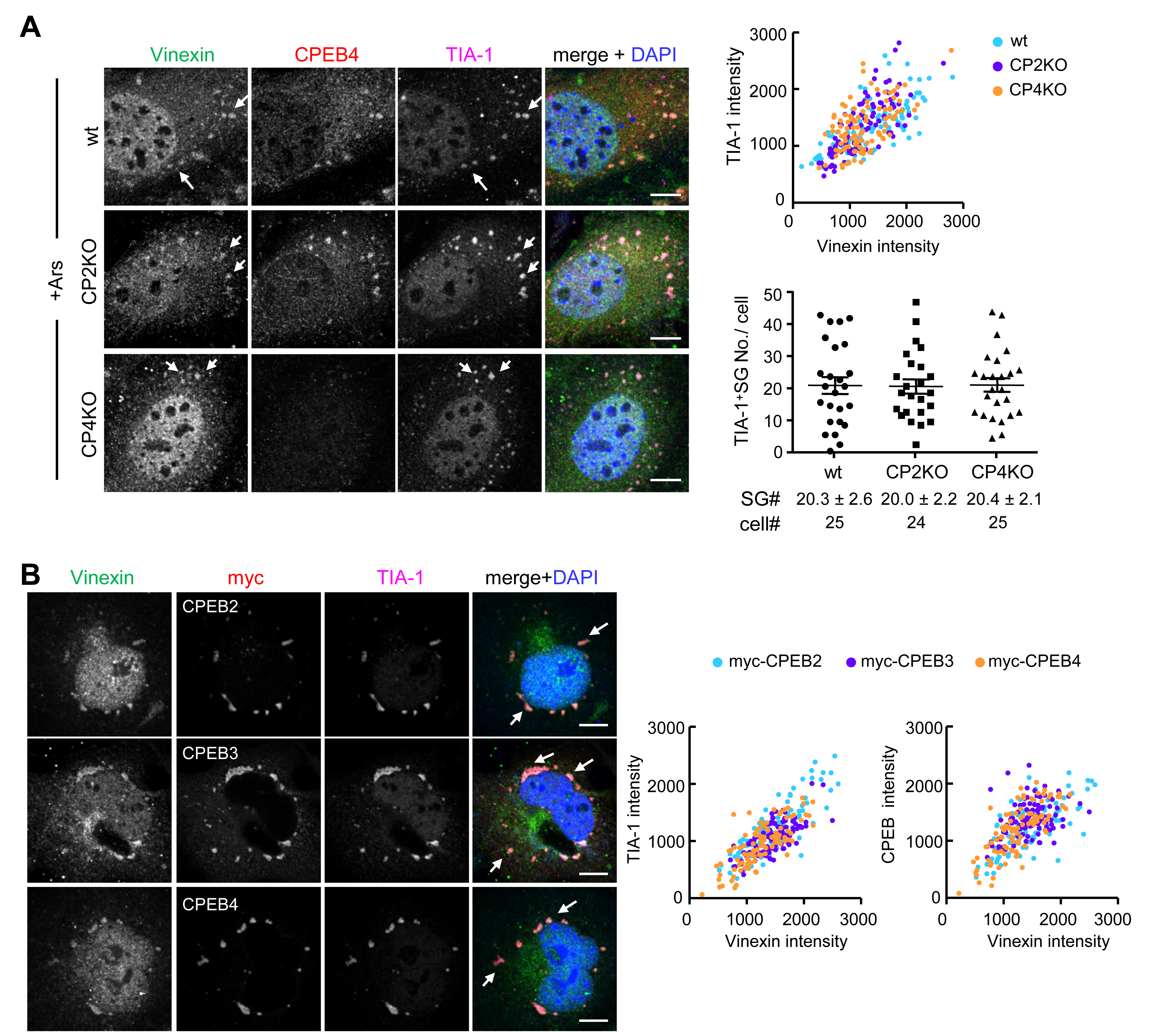

Supplement: Figure S6 — Overexpression of either one of CPEBs2-4 induces SG localization of Vinexin. (A) The wild-type (wt), CPEB2 knockout (CP2KO) and CPEB4 knockout (CP4KO) MEFs were treated with arsenite and then fixed for immunodetection of Vinexin, CPEB4 and TIA-1. Arrows indicate TIA-1-positive SGs. One hundred SGs were randomly selected in ten cell images taken from arsenite-treated wt or KO MEFs to quantify the signal intensities of Vinexin and TIA-1 in SGs. For each cell, the number of TIA-1-positive SGs was analyzed and displayed in the dot plot. The average SG number per cell (mean ± s.e.m.) and the number of analyzed cells are listed at the bottom. (B) COS-7 cells transfected with the plasmid expressing myc-tagged CPEB2, CPEB3 or CPEB4 were immunostained with Vinexin and TIA-1 antibodies. Arrows indicate SGs. The immunostained signal of Vinexin was plotted against that of TIA-1 or myc-CPEB in a hundred SGs randomly selected from ten transfected cells. Scale: 10 µm. (TIF) [file pone.0107961.s006.tif]

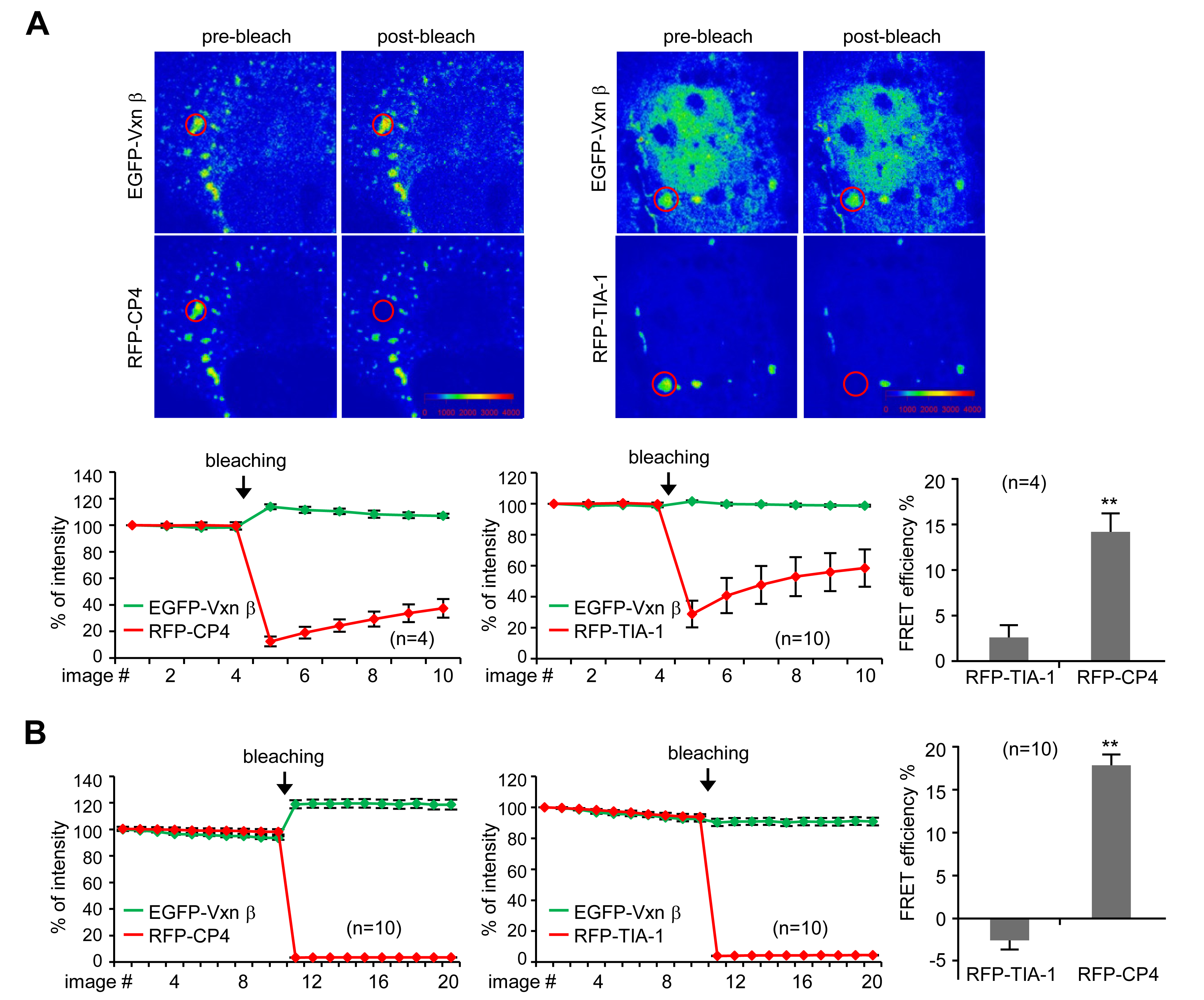

Supplement: Figure S7 — FRET detection of CPEB4-Vinexin interaction in SGs. The plasmids encoding the FRET donor EGFP-Vinexin (Vxn) β and acceptor RFP-CPEB4 (CP4) or RFP-TIA-1 were co-transfected to COS-7 cells. (A) The live cells were used for FRET analysis to detect the interaction of Vinexin with CPEB4 or TIA-1 in the selected SG (red circle). The example images show that the fluorescent signal of EGFP-Vxn β increases after photobleaching the acceptor RFP-CP4 but not RFP-TIA-1. The changes in fluorescence intensity of EGFP right before and after bleaching RFP were calculated as FRET efficiency. All of the data were expressed as the mean ± s.e.m. n: the number of SGs and cells in each group (one SG per cell was performed for FRET analysis). (B) Similar to (A), except the fixed samples were used for FRET analysis. (TIF) [file pone.0107961.s007.tif]
