# Supplementary material for: Arsenite-Activated JNK Signaling Enhances CPEB4-Vinexin Interaction to Facilitate Stress Granule Assembly and Cell Survival
Source: PLoS One. 2014 Sep 19;9(9):e107961. doi: 10.1371/journal.pone.0107961 (PMC4169592; doi:10.1371/journal.pone.0107961)
Supplement: Table S1 — The sequences of primers used for constructing CPEB4 and Vinexin mutants. (DOCX) [file pone.0107961.s008.docx]

**Table S1.** The sequences of primers used for constructing CPEB4 and Vinexin mutants

| Primer name | 5’ → 3’ sequence |
| --- | --- |
| rCPEB4 mut1F | ggtaccgagctcggatccaccgggcaggaagctggaatactg |
| rCPEB4 mut1R | cagtattccagcttcctgcccggtggatccgagctcggtacc |
| rCPEB4 mut2F | ggtaccgagctcggatccaccaataatggtgctctcttgtttcaaaatttc |
| rCPEB4 mut2R | gaaattttgaaacaagagagcaccattattggtggatccgagctcggtacc |
| rCPEB4 mut3F | ggtaccgagctcggatccaccccgggaggtggcggctat |
| rCPEB4 mut3R | atagccgccacctcccggggtggatccgagctcggtacc |
| rCPEB4 mut4F | ggtaccgagctcggatccaccattaaagcaaggacttatgggcgaag |
| rCPEB4 mut4R | cttcgcccataagtccttgctttaatggtggatccgagctcggtacc |
| rCPEB4 mut5F | gtccctggcgcttcagctccgggaggtggcggc |
| rCPEB4 mut5R | gccgccacctcccggagctgaagcgccagggac |
| rCPEB4 mut6F | ccccctcttcttcctggagcattaaagcaaggacttatgggcg |
| rCPEB4 mut6R | cgcccataagtccttgctttaatgctccaggaagaagaggggg |
| rCPEB4 mut7F | ggtccctggcgcttcagctattaaagcaaggacttatgggcgaag |
| rCPEB4 mut7R | cttcgcccataagtccttgctttaatagctgaagcgccagggacc |
| rCPEB4 ∆PRD1F | ggcctctctcccagcaccatcagcaccatcacagccagcatc |
| rCPEB4 ∆PRD1R | gatgctggctgtgatggtgctgatggtgctgggagagaggcc |
| rCPEB4 ∆PRD2F | gcatcagcagcagaggaggtctccacctttcacacatagaaatgctg |
| rCPEB4 ∆PRD2R | cagcatttctatgtgtgaaaggtggagacctcctctgctgctgatgc |
| rCPEB4 ∆PRD3F | cctcatttggcgaataatcttaacaaaagctaccagagtccctctccaacc |
| rCPEB4 ∆PRD3R | ggttggagagggactctggtagcttttgttaagattattcgccaaatgagg |
| rCPEB4 ∆PRD4F | gatagcttgaacagagctgacaacattaggacatttgacatgcactcattg |
| rCPEB4 ∆PRD4R | caatgagtgcatgtcaaatgtcctaatgttgtcagctctgttcaagctatc |
| mVinexin βNF | gccagctacgtgcagataaaccgatgagcggccgctcgagtctag |
| mVinexin βNR | ctagactcgagcggccgctcatcggtttatctgcacgtagctggc |
| mVinexin βCF | gacgatgatgacaagggcgaattcgagccccggctcaggctttgtgat |
| mVinexin βCR | atcacaaagcctgagccggggctcgaattcgcccttgtcatcatcgtc |
| mVinexin β∆(SH3)1F | gaagaggaaggccgcccgggttctgcctgccgatgagat |
| mVinexin β∆(SH3)1R | atctcatcggcaggcagaacccgggcggccttcctcttc |
| mVinexin β∆(SH3)2F | ggagtatggcgatgcagtaataaaccgagagccccggctc |
| mVinexin β∆(SH3)2R | gagccggggctctcggtttattactgcatcgccatactcc |
| mVinexin β∆(SH3)3F | gatacactggactccgtaccggtgagcggccgctcgagtctag |
| mVinexin β∆(SH3)3R | ctagactcgagcggccgctcaccggtacggagtccagtgtatc |
